# Inter-Relationship Between Melanoma Vemurafenib Tolerance Thresholds and Metabolic Pathway Choice

**DOI:** 10.3390/cells14120923

**Published:** 2025-06-18

**Authors:** Pratima Nangia-Makker, Madison Ahrens, Neeraja Purandare, Siddhesh Aras, Jing Li, Katherine Gurdziel, Hyejeong Jang, Seongho Kim, Malathy P Shekhar

**Affiliations:** 1 Karmanos Cancer Institute, 421 E. Canfield Avenue, Detroit, MI 48201, USA; makkerp@karmanos.org (P.N.-M.); ahrensma@msu.edu (M.A.); lijing@wayne.edu (J.L.); jangh@karmanos.org (H.J.); kimse@karmanos.org (S.K.); 2 Department of Oncology, Wayne State University School of Medicine, 421 E. Canfield Avenue, Detroit, MI 48201, USA; saras@wayne.edu; 3 Center for Molecular Medicine and Genetics, Wayne State University School of Medicine, 540 E. Canfield Avenue, Detroit, MI 48201, USA; npuranda@wayne.edu; 4 Institute of Environmental Health Sciences, Wayne State University, Detroit, MI 48202, USA; gurdziel@wayne.edu; 5 Department of Pharmacology, School of Medicine, Wayne State University, Detroit, MI 48202, USA; 6 Department of Pathology, Wayne State University School of Medicine, 421 E. Canfield Avenue, Detroit, MI 48201, USA

**Keywords:** melanoma, BRAF, adaptive resistance, RNA-seq, metabolome, Wnt signaling

## Abstract

Melanomas quickly acquire resistance to vemurafenib, an important therapeutic for BRAFV600 mutant melanomas. Although combating vemurafenib resistance (VemR) to counter mitochondrial metabolic shift using mitochondria-targeting therapies has promise, no studies have analyzed the relationship between vemurafenib tolerance levels and metabolic plasticity. To determine how vemurafenib endurance levels drive metabolic plasticity, we developed isogenic BRAFV600E VemR melanoma models with variant vemurafenib tolerances and performed an integrative analysis of metabolomic and transcriptome alterations using metabolome, Mitoplate-S1, Seahorse, and RNA-seq assays. Regardless of drug tolerance differences, both VemR models display resistance to MEK inhibitor and sensitivity to Wnt/β-catenin inhibitor, ICG-001. β-catenin, MITF, and ABCB5 levels are upregulated in both VemR models, and ICG-001 treatment restored vemurafenib sensitivity with reductions in MITF, ABCB5, phospho-ERK1/2, and mitochondrial respiration. Whereas β-catenin signaling induced TCA cycle and OXPHOS in highly drug tolerant A2058VemR cells, it activated pentose phosphate pathway in M14VemR cells with low vemurafenib tolerance, both of which are inhibited by ICG-001. These data implicate an important role for Wnt/β-catenin signaling in VemR-induced metabolic plasticity. Our data demonstrate that drug tolerance thresholds play a direct role in driving metabolic shifts towards specific routes, thus providing a new basis for delineating VemR melanomas for metabolism-targeting therapies.

## 1. Introduction

Malignant melanoma is a deadly form of skin cancer, with exposure to ultraviolet radiation being a major risk factor for melanoma development. The 5-year survival rate for patients with metastatic melanoma is less than 25% [1]. BRAF, a serine-threonine protein kinase, acting in the RAS-RAF-MEK-ERK signaling pathway, is mutated in approximately 65% of malignant cutaneous melanomas, and >90% of the cases involve V600 mutations [2]. Approximately 15–20% of melanomas have mutations in *NRAS*, while ~6% have normal BRAF [3]. Vemurafenib and dabrafenib are FDA-approved BRAF inhibitors (BRAFi) for treating non-resectable melanomas with *V600E/K BRAF* mutation. Despite high initial response rates, acquired resistance to these drugs is common, and most patients relapse within months. The advent of immune checkpoint inhibitors (ICIs) has produced longer-lasting responses; however,  > 75% of patients experience limited therapeutic benefit, and initial ICI-responders develop disease progression [4,5]. Emerging evidence supports combining RAS–RAF-ERK pathway inhibitors such as vemurafenib with ICIs for improving therapeutic response, indicating that BRAFi(s) continue to constitute an important armamentarium for metastatic melanoma treatment. Resistance to BRAFi(s) is linked to nonmutational adaptation of melanoma cells to the drug, which is initiated during the early phase of treatment and continues through the tolerant phase of drug treatment [6]. This acclimatization could provide ample opportunities for triggering or resetting pathways that promote BRAFi insensitivity and alterations in melanoma metabolism.

Melanoma malignancy is associated with elevated glycolytic activity and lower mitochondrial respiration even under normoxic conditions [7], which is referred to as the Warburg effect, a hallmark of many cancer types [8]. *BRAFV600E* mutation is associated with metabolic reprogramming via induction of ERK1/2 hyperactivation and promotion of glycolytic enzyme enrichments [9,10]. As an adaptive response to combat drug-induced metabolic stress, BRAFi(s) reverse the Warburg effect, thus reducing glycolytic activity and stimulating mitochondrial biogenesis through the MITF-PGC1α pathway [11,12]. Accumulating evidence links altered metabolism with Wnt signaling, and aberrant activation of both the canonical (β-catenin dependent) and noncanonical (β-catenin independent) mechanisms of Wnt signaling is associated with neoplastic proliferation, metastatic dissemination, and therapy resistance [13,14]. Canonical Wnt/β-catenin signaling directly regulates transcriptional activation of MITF, a lineage-specific transcription factor critical for melanocyte differentiation [15], and studies show that Wnt/β-catenin, MAPK/ERK, and PI3K/AKT signaling pathways converge to regulate nuclear transport and transcriptional activity of MITF [16]. This suggests that canonical Wnt signaling may directly contribute to metabolic mitochondrial reprogramming via regulation of MITF-mediated effects on mitochondrial biogenesis in BRAF mutant melanomas.

Metabolic plasticity is a major factor in melanoma drug resistance and aggressiveness. Thus, understanding the molecular mechanisms driving metabolic adaptations is crucial for developing metabolic pathway-specific therapies. Melanoma patients, despite having BRAF mutation, show varied responses to vemurafenib; however, no studies to date have analyzed the molecular relationships between vemurafenib tolerance thresholds and the specific metabolic pathways utilized to ensure melanoma survival. To determine whether vemurafenib endurance levels contribute to metabolic plasticity, we established BRAF mutant isogenic models of human metastatic melanoma with variant levels of VemR and performed an integrated analysis of the metabolic and transcriptome alterations induced by adaptive VemR. Regardless of drug tolerance differences, the VemR models show upregulation of Wnt/β-catenin signaling and resistance to MEK inhibitors. Treatment with β-catenin inhibitor ICG-001 restored vemurafenib sensitivity with reductions in MITF, ABCB5, and phospho-ERK1/2 expressions. Targeted metabolome, MitoPlate S-1 and Seahorse assays support a critical role for β-catenin signaling in activation of mitochondrial metabolism and OXPHOS in melanomas with high drug tolerance such as A2058VemR cells, whereas melanomas such as M14VemR cells with low vemurafenib tolerance threshold cells use β-catenin signaling for activating the pentose phosphate pathway (PPP), a critical metabolic route for nucleotide synthesis. Pathway analysis of transcripts showed that pathways associated with cytokine–cytokine receptor interaction, ECM receptor interaction, and neuroactive ligand receptor interaction were similarly enriched in patient-derived metastatic BRAF mutant Mel 14-108 melanoma as in M14 and A2058 models, whereas pathways associated with the cell cycle, DNA replication, Fanconi anemia, and DNA repair pathways were enriched in wild-type BRAF Mel 14-089 patient-derived melanoma, revealing distinct regulations by mutant and wild-type BRAF. Patient-derived Mel 14-108 cells have comparable vemurafenib sensitivity profiles as M14 cells and show similar vemurafenib-induced metabolic pathway perturbations of PPP, supporting the relationship between low vemurafenib tolerance and PPP activation. These data reveal a direct relationship between vemurafenib tolerance thresholds and metabolic pathway choices made by melanoma cells and imply the importance of understanding this relationship as it could provide a new rationale for delineating VemR melanomas for selective metabolism-targeting therapies.

## 2. Materials and Methods

### 2.1. Cell Lines and Cell Culture

Human metastatic melanoma cells A2058 and M14 were obtained from the American Type Culture Collection (ATCC, Manassas, VA, USA) and the National Cancer Institute (Bethesda, MD, USA), respectively. A2058 and M14 cells were cultured in Dulbecco’s modified Eagle’s medium (DMEM)/F12 medium supplemented with 5% fetal bovine serum at 37 °C, 5% CO_2_ [17]. The authenticated cell lines were used within 5–10 passages. Isogenic cells resistant to vemurafenib were generated by using a pulsed treatment strategy. M14 or A2058 cells were exposed to vemurafenib at their respective IC50 starting doses of 10 or 250 nM. Cells were treated for several days and then allowed to recover in drug-free medium. Doses were gradually increased with similar pulsed on–off rounds. Cell survival was assessed every 3–5 weeks to confirm sustained and increased tolerance thresholds for vemurafenib compared to their parental counterparts, and IC50 values were determined regularly. Vemurafenib-resistant (VemR) M14 or A2058 cells were maintained in DMEM/F12 medium supplemented with 5% FBS containing 100 nM or 5 µM vemurafenib, respectively. Patient-derived brain metastatic melanoma cells Mel 14-089 and Mel 14-108 were grown in DMEM/F12 media supplemented with 10% fetal bovine serum, non-essential amino acids, and gentamicin (Millipore Sigma, St. Louis, MO, USA) at 37 °C, 5% CO_2_. The details of Mel 14-089 and Mel 14-108 isolation from metastatic brain tumors were previously described [18]. Whereas Mel 14-108 cells express *BRAF V600E*, Mel 14-089 cells express wild-type BRAF. Acquisition and use of patient-derived samples were approved by the Wayne State University Institutional Review Board, and written informed consent was obtained from each patient prior to enrollment (IRB 111610MP2E; Protocol # 1011009008).

### 2.2. Cell Survival and Colony-Forming Analyses

Metastatic melanoma cells M14, A2058, their VemR counterparts M14 VemR and A2058 VemR, and patient-derived Mel 14-089 and Mel 14-108 cells were seeded at 7500 cells/well in 96-well plates in quadruplicates and treated with 0–10 µM vemurafenib or ICG-001 (Selleck Chemicals, Houston, TX, USA). Sensitivities of parental and VemR M14 and A2058 cells to MEK (U0126 or PD098059) or PI3K/Akt (LY294002) inhibitors were tested at 0–50 µM concentrations. Cell viability was assessed at 72 h post treatment by MTT assays. Experiments were performed in triplicate, and the results presented are representative of three independent experiments. For evaluation of colony-forming potentials, parental M14 and A2058 cells were treated overnight with 0–100 nM or 0–500 nM vemurafenib, respectively, and M14 VemR and A2058 VemR cells were treated with 100 nM or 5 µM vemurafenib, respectively. Cells were trypsinized and reseeded in quadruplicate at 250 cells/well in 24-well plates in drug-free media. Colonies were allowed to form for two to three weeks and detected by crystal violet staining. Colonies were quantified using GelCount™ Oxford Optronix and the CHARM algorithm (Oxford Optronics, Ltd., London, ON, Canada), with a minimum diameter of 100 µm set as the threshold for colony classification. Colony-forming efficiencies were expressed relative to the untreated control cells.

### 2.3. Western Blot Analysis 

Whole cell lysates were prepared as previously described [17] from parental and VemR M14 and A2058 cells, and patient-derived Mel 14-089 or Mel 14-108 cells treated with vemurafenib or vehicle. Approximately 50–100 µg of protein were subjected to 4–20% gradient SDS-PAGE and Western blot analysis of phospho-ERK1/2 (Cell Signaling, Danvers, MA, USA), ERK1/2 (Cell Signaling), Ser473phospho-AKT (Cell Signaling), AKT (Cell Signaling), β-catenin (Santa Cruz Biotechnology, Dallas, TX, USA), Melan A (Dako Corp., Santa Clara, CA, USA), vimentin (Dako Corp.), MITF (Abcam, Cambridge, MA, USA), total OXPHOS human antibody cocktail (Abcam, Waltham, MA, USA), Tomm 20 (Santa Cruz Biotechnology), ABCB5 (Abcam), β-actin, and α-tubulin (Sigma-Aldrich Chemicals, St. Louis, MO, USA). Protein levels relative to the loading control α-tubulin or β-actin were quantified by ImageJ version 1.53 (NIH, Bethesda, MD, USA). 

### 2.4. Chemotaxis Analysis

A2058 VemR cells were treated overnight with 5 µM vemurafenib alone or in combination with 0–10 µM ICG-001 and subjected to Boyden chamber chemotaxis assays as previously described [17]. Cells that migrated across the membrane were fixed and stained using the Diff-Quik stain set (Baxter, Deerfield, IL, USA). The staining intensities were quantified using ImageJ version 1.53.

### 2.5. Dual-Luciferase Reporter Assay

To evaluate the transcriptional activity of endogenous TCF/β-catenin, parental and VemR M14 and A2058 cells were transiently cotransfected with 1.0 µg of pTOP/FLASH or pFOP/FLASH (Upstate Biotech, Lake Placid, NY, USA) and 100 ng of Renilla luciferase pRL-TK (Promega, Madison, WI, USA) using Metafectene (Biontex Laboratories GmbH, Munich, Germany). Luciferase activities were measured as previously described using the Promega Dual-Luciferase Assay system [19]. Absolute promoter firefly luciferase activity was normalized against Renilla luciferase activity to correct for transfection efficiency. Triplicate dishes were assayed for each transfection, and at least three transfection assays were performed.

### 2.6. Mitochondria Imaging Analysis

Parental and VemR M14 or A2058 cells cultured on coverslips were treated overnight with vemurafenib (M14, 10 nM; M14 VemR, 100 nM; A2058, 250 nM; A2058 VemR, 5 µM) alone or in combination with 5 µM ICG-001. Cells were washed with serum-free media before incubation with 50 nM Mitotracker DeepRed FM (Molecular Probes, Eugene, OR, USA), a mitochondrial membrane potential-dependent dye, at 37 °C for 30 min. Cells were washed, fixed, and mounted in Slowfade containing 4′,6-diamidino-2-phenylindole (DAPI) to counterstain nuclei. Images were captured on an Olympus BX40 microscope equipped with a Sony high-resolution/high-sensitivity CCD video camera and CellSens V4.4 software. The percentage of cells with active mitochondria and the integrated staining densities reflecting active mitochondrial mass were determined using the ImageJ tool and scored from at least 30–50 cells/field and three to five fields.

### 2.7. Analysis of Sugar Utilization Flexibility

To compare sugar utilization flexibility and survival of parental and VemR M14 or A2058 cells, cells were cultured in media containing high or low glucose or galactose. Dialyzed fetal bovine serum (5%) was used for all experiments. The compositions of the media were as follows: high-glucose medium: DMEM deprived of glucose (Cat.# A14430; Invitrogen, Carlsbad, CA, USA) supplemented with 25 mM D-glucose, 0.5 mM sodium pyruvate, 2.5 mM L-glutamine, 5% FBS, and penicillin-streptomycin (500 µg/mL final concentration); low-glucose medium: DMEM deprived of glucose supplemented with 5 mM D-glucose, 0.5 mM sodium pyruvate, 2.5 mM L-glutamine, 5% FBS, and penicillin-streptomycin; galactose medium: DMEM deprived of glucose supplemented with 25 mM D-galactose, 0.5 mM sodium pyruvate, 2.5 mM L-glutamine, 5% FBS, and penicillin-streptomycin. Prior to the experiment, cells cultured in high-glucose DMEM/F12 media were washed with PBS, followed by incubation for 8–10 h in sugar-free medium to facilitate glucose depletion. Cells were trypsinized, pelleted, and rinsed thrice with PBS. After washing, cell pellets were resuspended in high-glucose, low-glucose, or galactose media, and seeded at 2 × 10^4^ cells/well in quadruplicates in 96-well plates. Cell viability was measured at 72 to 96 h by MTT assay, and the results were expressed as mean ± S.D., relative to the corresponding high-glucose condition. At least three independent assays were performed. To determine the functional involvement of β-catenin on sugar utilization, M14 and A2058 VemR cells were seeded under high-glucose and galactose conditions in 96-well plates and treated with 1–10 µM ICG-001. Cell viability was measured at 72 to 96 h by MTT assay as described above.

### 2.8. Cellular Bioenergetics Analysis

Oxygen consumption rates (OCR) of parental and VemR M14 and A2058 cells cultured in glucose- or galactose-containing media or treated with ICG-001 were measured using the XFe24 flux analyzer and the Cell Mito Stress Test kit (Agilent Technologies, Santa Clara, CA, USA) according to the manufacturer’s instructions. Cells were seeded at a density of 3 × 10^4^ cells/well in quintuplicates in Seahorse XFe24 24-well plates at 37 °C. Global mitochondrial parameters, i.e., basal respiration, ATP production, maximal respiratory capacity, and non-mitochondrial respiration, were determined by sequential injection of ATPase inhibitor oligomycin (1 μM), an uncoupler of the respiratory chain carbonyl cyanide-4-(trifluoromethoxy)phenylhydrazone (FCCP, 1 μM), and ETC complex I and III inhibitors, rotenone (0.5 μM) and antimycin A (0.5 μM), respectively. Data were analyzed using the XF Wave Desktop Software (Agilent Technologies).

### 2.9. Immunofluorescence Staining

M14 VemR and A2058 VemR cells were seeded on coverslips and treated overnight with 100 nM or 4 µM vemurafenib, respectively, or in combination with 5 µM ICG-001. Cells were fixed with 10% phosphate-buffered formalin, permeabilized with methanol/acetone (1:1, *v*/*v*), and immunostained with β-catenin antibody (Santa Cruz Biotechnology) and corresponding Texas Red-conjugated secondary antibody (Molecular Probes, Eugene, OR, USA). Nuclei were counterstained with DAPI. Images were collected on an Olympus BX60 microscope equipped with a Sony high-resolution/high-sensitivity CCD video camera and processed using CellSens V4.4 software.

### 2.10. Targeted Metabolomics

Steady-state levels of metabolites in parental and VemR M14 and A2058 cells were quantitatively profiled using an established LC-MS/MS-based targeted metabolomics platform, which measures 254 metabolites involved in major human metabolic pathways [20]. All LC-MS/MS analyses were performed on an AB SCIEX QTRAP 6500 LC-MS/MS system, which consists of a SHIMADZU Nexera ultra-high-performance liquid chromatography coupled with a triple quadrupole/linear ion trap mass spectrometer. Analyst 1.6 software was used for system control and data acquisition, and MultiQuant 3.0 software was used for data processing and quantitation. Metabolites in parental and VemR M14 and A2058 cells were extracted with ice-cold 80% methanol and subjected to LC-MS/MS analyses as previously described [20]. Metabolite concentrations were normalized to cellular protein. Statistical analysis and pathway analysis were conducted using MetaboAnalyst (www.MetaboAnalyst.ca, version 6.0) [21]. Comparisons of individual metabolites between the groups were performed using unpaired t-tests with FDR correction applied using the Benjamini–Hochberg method.

### 2.11. Mitochondrial and Cytosol Metabolism Analysis

Alterations in metabolism inside the mitochondria and/or cytosol induced by acquired vemurafenib resistance were evaluated using the MitoPlate S-1 assay (Biolog Inc., Hayward, CA, USA). MitoPlate S-1 plate contains 3 sets of precoated 31 substrates of mitochondrial or glycolytic metabolism, plus controls, and allows for interrogation and characterization of substrate metabolism rates. The assay was performed according to the manufacturer’s directions. Briefly, the precoated wells were incubated for 1 h at 37 °C with 30 µL of Biolog mitochondrial assay solution (MAS) containing redox dye MC (Biolog Inc.) and 100 µg/mL saponin. Parental and VemR M14 or A2058 cells were resuspended in Biolog MAS at 1 × 10^6^ cells/mL, and 30 µL of cell suspension was added to the preincubated wells. In some cases, cells were treated with 1 µM ICG-001 to determine the effect of β-catenin activity inhibition on mitochondrial and cytoplasmic metabolism. MitoPlates were immediately loaded into BioTek Synergy2 microplate reader to perform kinetic measurements at 590 nm at 10 min intervals for 4 h at 37 °C. Data were normalized using the “No substrate” well (A1) required for rows A to H (cytoplasmic substrates, rows A and B; mitochondrial substrates, rows C–H). Data were analyzed using R, which creates a scatterplot of the initial rate values between conditions of interest.

### 2.12. Whole Genome Expression Analysis by RNA-Seq

To identify transcripts that are affected by VemR acquisition, parental M14 and A2058 cells and their VemR-resistant counterparts were subjected to bulk RNA-seq analysis. To determine the impact of BRAF mutation on the transcriptome, metastatic melanoma PDXs with BRAF mutation (Mel 14-108) or wild-type BRAF (Mel 14-089) were treated with 10 nM or 4 µM vemurafenib, respectively, and similarly subjected to bulk RNA-seq analysis. Total RNA was isolated using the Trizol reagent kit (Invitrogen, Carlsbad, CA, USA), and RNA-seq libraries were prepared using Lexogen’s QuantSeq 3′mRNA-seq Library Prep Kit (FWD for Illumina) from 200 ng of DNase I-treated RNA. The barcoded libraries were sequenced on an Illumina NovaSeq 6000. RNA-seq analysis was conducted through the Genome Sciences Core at Wayne State University. After data were demultiplexed using Illumina’s CASAVA 1.8.2 software, reads were aligned to the human reference genome (Build hg38) [22] and tabulated before analysis with R/Bioconductor package edgeR (version 3.36.0) [23]. For differential gene expression analysis, the edgeR function ‘glmQLFTest’ was used. FDR was computed using the Benjamini–Hochberg method [24], and heatmap and hierarchical clustering were carried out using the R package Complex Heatmap (version 2.22). Differentially expressed genes (DEGs) between parental and VemR counterparts were detected using 5% FDR and fold-change (FC) of ≥1.5. iPathwayGuide Analysis (Advaita Bioinformatics, Ann Arbor, MI, USA) was performed to obtain biological information on the pathways perturbed by vemurafenib resistance acquisition.

### 2.13. Real-Time Reverse Transcriptase (RT)-PCR Analysis

The sequencing data were validated by quantitative RT-PCR analysis of β-catenin, MITF-M, TYRP1, ABCB5, RBKS, and GAPDH reference control expressions in M14 VemR and A2058 VemR cells using DNase I-treated total RNAs and Maxima SYBR Green/ROX qPCR mix (ThermoFisher, Waltham, MA, USA). Primers for real-time RT-PCR analysis were as follows: β-catenin (accession number NM_001098209), 5′-ATACCACCCACTTGGCAGAC-3′ (forward) and 5′-GGAAGGTCTCCTTGGGACTC-3′ (reverse); TYRP1 (accession number NM_000550), 5′-ATGGCAACACGCCACAATTTGAG-3′ (forward) and 5′-CCCGTTGCAAAATTCCAGTAAG-3′ (reverse); MITF-M (MITF transcript isoform 4; accession number NM_000248), 5′-ATGCTGGAAATGCTAGAATATAATCACTATCAG-3′ (forward) and 5′-AGCCATGGGGCTGTTGGGTGC-3′ (reverse); RBKS (accession number BC017425.1), 5′-GCCGAGCCAAAGTGATGATATG-3′ (forward) and 5′-TGGAGAGGGTATAGAACTGGGG-3′ (reverse); ABCB5 (accession number MK803370), 5′-GCTGAGGAATCCACCCAATCT-3 ’ (forward) and 5′-CACAAAAGGCCATTCAGGCT-3′ (reverse), and GAPDH (accession number NM_002046), 5′-AAATATGATGACACCAAGAAGG-3′ (forward) and 5′-TGAAGTCGGAGGAGACCAC-3′ (reverse). Amplification was performed in StepOne Plus Real-Time PCR system (Applied Biosystems, Foster City, CA, USA), and the cycling conditions were 10 min at 95 °C, 35 cycles of 15 s at 95 °C, and 1 min at 57 °C, and 1 min at 68 °C.

### 2.14. Statistical Analysis

Comparisons between two groups were performed using unpaired t-tests, and for three or more groups, ANOVA was utilized along with Holm’s post hoc analysis. Differential analyses for metabolomics and RNA-seq data were performed using unpaired t-tests or moderated t-tests, followed by FDR correction. The volcano plot was generated with FDR < 0.05 and fold-change > 1.5. Multi-omics analysis was conducted between metabolomics and RNA-seq data, with significant pathways identified using metabolomics data. For each significant pathway, genes that were present in the pathway were extracted to construct a gene set. For each of the gene sets, heatmaps were generated, followed by gene set enrichment analysis (GSEA). Experimental results are presented as the mean ± standard deviation (S.D.) or standard error of mean (S.E.M). Statistical significances were considered if *p* < 0.05. All statistical analyses were performed with R (version 4.4.1), GraphPad Prism 4, or Microsoft Excel (Microsoft 365, version 2202).

## 3. Results

### 3.1. Generation and Characterization of Isogenic Models of Vemurafenib Resistant (VemR) Melanoma Cells

**Figure 1:**
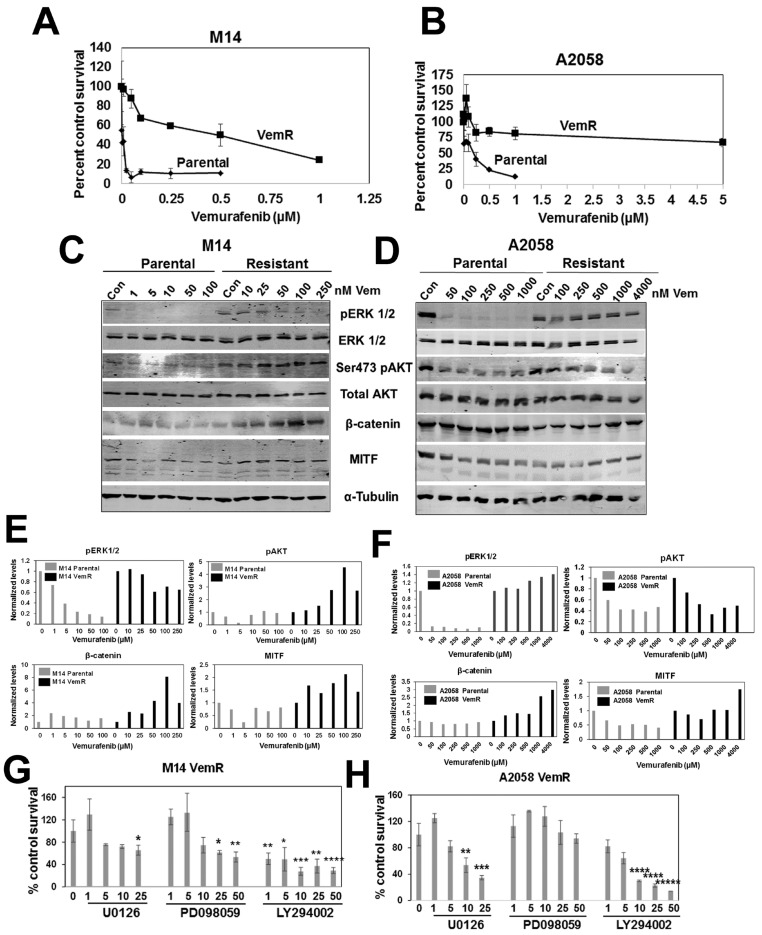
Characterization of isogenic melanoma models of vemurafenib resistance. (**A**,**B**) MTT assays of isogenic pairs of parental and vemurafenib-resistant (VemR) M14 and A2058 cells. (**C**,**D**) Western blot analysis of the indicated proteins following treatment with the indicated doses of vemurafenib, and (**E**,**F**) quantification of the indicated protein levels from representative blots of M14 (**E**) and A2058 (**F**) isogenic pairs. (**G**,**H**) Cell survival analysis of M14 VemR (**G**) and A2058 VemR (**H**) treated with MEK (U0126, PD098059) and PI3K (LY294002) inhibitors by MTT assay. Results are expressed as mean ± S.D. (percent of control cell survival) from three independent experiments. * *p* < 0.05; ** *p* < 0.01; *** *p* < 0.001; **** *p* < 0.0001; ***** *p* < 0.00001.

The melanoma lines, M14 and A2058, were chosen as they both carry the *BRAF V600E* mutation [25]. MTT assays showed that the M14 cell line is more responsive to vemurafenib treatment compared to A2058 cells, with IC50 values of 10 nM and 250 nM, respectively (Figure 1A,B). The results of MTT assays were further verified by colony-forming assays. Colony-forming potentials of M14 and A2058 cells were significantly inhibited with 5 nM (*p* < 0.001) and 50 nM (*p* < 0.01) vemurafenib (Figure S1A–D). Clinical trials using vemurafenib and other selective BRAFi(s) have shown impressive results; however, despite initial therapeutic responses, the development of acquired or adaptive resistance limits their clinical efficacy. To develop isogenic models of VemR, cells were exposed to gradually increasing concentrations of vemurafenib over several weeks. Cell survival was assessed every 3–5 weeks to verify increased vemurafenib tolerance thresholds compared to their parental counterparts, and IC_50_ values were determined regularly. MTT assays confirmed the increased abilities of vemurafenib-adapted M14 and A2058 cells to tolerate vemurafenib, with M14 VemR and A2058 VemR cells displaying IC50s of 0.1 µM and 5 µM, respectively (Figure 1A,B). M14 and A2058 VemR cells were continuously maintained in medium containing 100 nM or 5 µM vemurafenib, respectively.

To establish whether the differences in vemurafenib sensitivities between the corresponding isogenic pairs correlated with differences in inhibition of ERK kinase, a direct downstream effector of BRAF, we evaluated the expression levels of phospho-ERK1/2. Whereas ERK1/2 phosphorylation was inhibited by vemurafenib treatment in parental M14 and A2058 lines (Figure 1C,D), phospho-ERK1/2 levels remained largely unaffected by vemurafenib in the resistant counterparts (Figure 1C–F). Expression levels of Akt Ser473 phosphorylation were analyzed since PI3K/AKT pathway is implicated in VemR. Phospho-Ser473 Akt levels were upregulated or unaffected by VemR in M14 VemR or A2058 VemR cells, respectively, compared to their parental controls (Figure 1C–F).

Activation of Wnt/β-catenin signaling and MITF, a β-catenin transcriptional target, are implicated as drivers of BRAFi resistance in cancer cells carrying the *BRAFV600E* mutation [26,27]. To determine whether BRAF/Wnt/β-catenin crosstalk plays a role in adaptive VemR, we analyzed vemurafenib effects on β-catenin and MITF levels. While β-catenin levels were unaffected by vemurafenib in both M14 and A2058 parental lines (Figure 1C–F), treatment with ≥50 nM or ≥1 µM vemurafenib induced 2–4-fold increases in β-catenin levels in M14 VemR and A2058 VemR cells, respectively (Figure 1C–F). MITF protein levels were increased ~2-fold by vemurafenib in VemR models, mirroring β-catenin profiles (Figure 1C–F).

To further confirm the relevance of ERK and Akt activities in VemR, we analyzed the effects of MEK (U0126 and PD098059) and PI3K/Akt (LY294002) inhibitors on M14 VemR and A2058 VemR cell survival by MTT assays. Compared to parental M14 and A2058 cells that displayed IC50 values of 0.6 µM and 4 µM, respectively, for U0126 (Figure S2A,B), both VemR models were less sensitive to MEK inhibitors, requiring 25 µM PD098059 and >10 µM U0126 to induce 50% inhibition of cell survival (Figure 1G,H). Both parental and VemR M14 and A2058 cells responded similarly to LY294002 treatment with IC50s of ~1.5 µM and ~6.5 µM, respectively, suggesting only a minor role for PI3K/Akt activity in our models (Figure 1G,H and Figure S2A,B). Taken together, these data establish the validity of our VemR isogenic models and implicate a potential role for ERK and β-catenin signaling crosstalk in VemR.

### 3.2. β-Catenin Transcriptional Activity Inhibition Sensitizes Vemurafenib Resistant Melanoma Cells

**Figure 2:**
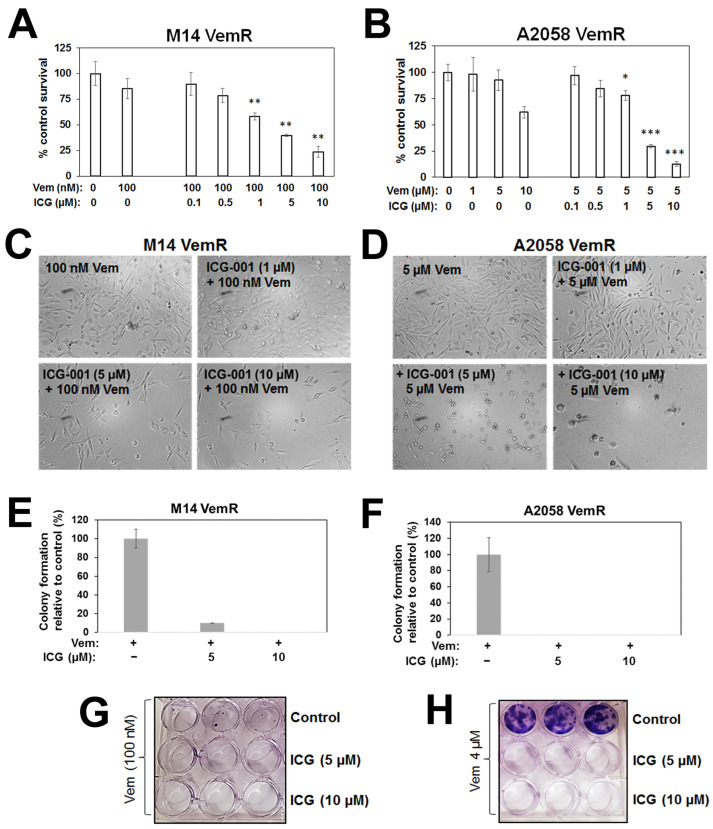
Sensitivity analysis of β-catenin inhibitor ICG-001 on vemurafenib sensitivities of M14 and A2058 VemR cells. (**A**,**B**) Evaluation of ICG-001 effects on survival of M14 VemR cells exposed to 100 nM vemurafenib (**A**) or A2058 VemR cells exposed to 5 µM vemurafenib (**B**) for 72 h by MTT assays, and (**C**,**D**) corresponding phase–contrast micrographs. (**E**,**F**) ICG-001 effects on colony-forming potentials of M14 VemR (**E**) and A2058 VemR (**F**) cells. Results are expressed as mean ± S.D. (percent of control colony formation efficiency) from three independent experiments. * *p* < 0.05; ** *p* < 0.01; *** *p* < 0.001. (**G**,**H**) Images of representative colonies captured using GelCount Oxford Optronix (Oxford Optronics, Ltd., London, ON, Canada).

To determine if activation of β-catenin signaling contributes to increased tolerance to vemurafenib, we analyzed the effect of β-catenin transcriptional inhibitor, ICG-001, on vemurafenib sensitivity by MTT and colony-forming assays. M14 VemR and A2058 VemR cells cultured in the presence of 100 nM or 5 µM vemurafenib, respectively, were treated with 0.1–10 µM ICG-001. Treatment with ICG-001 significantly suppressed proliferation of both M14 VemR (*p* < 0.01) and A2058 VemR (*p* < 0.05) cells (Figure 2A,B), and phase-contrast microscopy confirmed ICG-001-induced cytotoxicity (Figure 2C,D). Treatment with 5 or 10 µM ICG-001 abrogated M14 VemR and A2058 VemR colony formation, verifying the MTT assay results (Figure 2E–H). Treatment with ML329, a small molecule inhibitor of MITF, significantly inhibited M14 VemR and A2058 VemR cell survival (*p* < 0.01), supporting the role of MITF (a β-catenin transcriptional target) and β-catenin activity in VemR (Figure S3).

**Figure 3:**
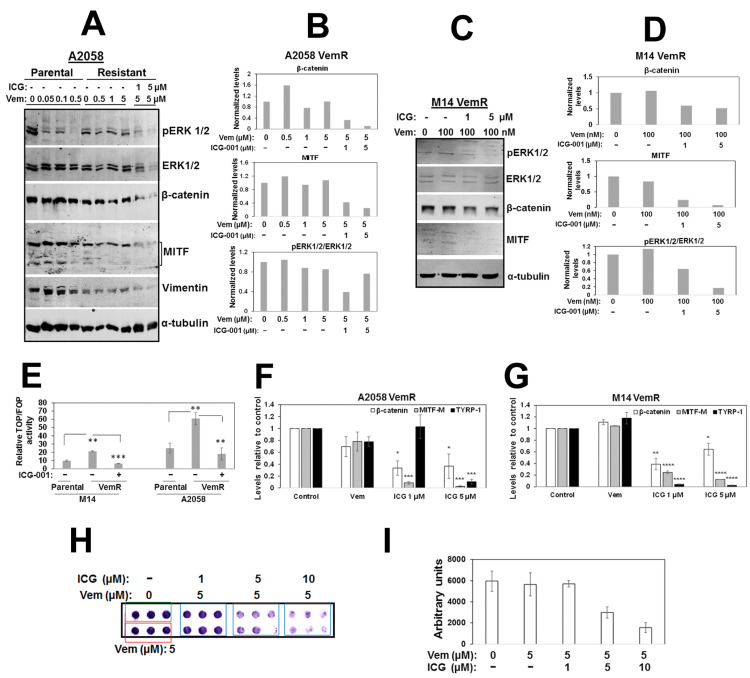
ICG-001 treatment downregulated β-catenin-mediated transcriptional activity and β-catenin-regulated genes. (**A**,**C**) Western blot analysis of the indicated proteins following overnight treatment with the indicated concentrations of vemurafenib or ICG-001 in A2058 VemR (**A**) or M14 VemR (**C**) cells, and (**B**,**D**) relative levels of the indicated proteins in A2058 VemR (**B**) and M14 VemR (**D**) cells. (**E**) β-catenin transcriptional activity and ICG-001 regulation in parental and VemR M14 and A2058 isogenic cells. (**F**,**G**) Real-time quantitative RT-PCR analysis of β-catenin-regulated gene expression in A2058 VemR (**F**) and M14 VemR (**G**) cells. (**H**) ICG-001 effects on A2058 VemR cell migration using Boyden chamber. The green and red colored boxes show migration of A2058 cells (in triplicates) treated with vehicle or 5 µM vemurafenib, respectively. All other treatments were done in sextuplicates. (**I**) quantitation using Image J software. * *p* < 0.05; ** *p* < 0.01; *** *p* < 0.001; **** *p* < 0.0001.

To determine whether ICG-001-induced sensitization of VemR cells resulted from the combined inhibition of ERK and β-catenin signaling, we analyzed the effects of ICG-001 on phospho-ERK1/2, ERK1/2, β-catenin, MITF, and vimentin (also a β-catenin transcriptional target) protein levels. Treatment with ICG-001 elicited noticeable decreases in phospho-ERK1/2, β-catenin, MITF, and vimentin in VemR cells compared to cells treated with vemurafenib alone (Figure 3A–D). ICG-001 treatment also reduced total ERK1/2 levels in A2058 VemR cells, suggesting a potential impact of the β-catenin inhibitor on ERK1/2 stability (Figure 3A). TOP/Flash reporter assays showed ~2–3-fold increases, respectively, in β-catenin-dependent transcriptional activity in M14 VemR and A2058 VemR cells compared to their respective parental counterparts (*p* < 0.01), and treatment with ICG-001 significantly decreased TOP/Flash activity in M14 VemR (*p* < 0.001) and A2058 VemR (*p* < 0.01) cells (Figure 3E). Consistent with TOP/Flash reporter data, real-time RT-PCR analysis showed ICG-001 significantly decreased expressions of β-catenin (*p* < 0.05), MITF-M (*p* < 0.001), and TYRP1 (*p* < 0.001) in both VemR models (Figure 3F,G). Boyden chamber assays showed that ICG-001 treatment decreased A2058 VemR cell migration in a dose-dependent manner compared to cells treated with vemurafenib alone (Figure 3H,I), corroborating β-catenin involvement in migration and invasion. Taken together, these data reveal a major role for Wnt/β-catenin signaling in persistent activation of ERK1/2 in VemR cells, and that inhibition of β-catenin transcriptional activity can disrupt this crosstalk and suppress BRAFi resistance.

**Figure 4:**
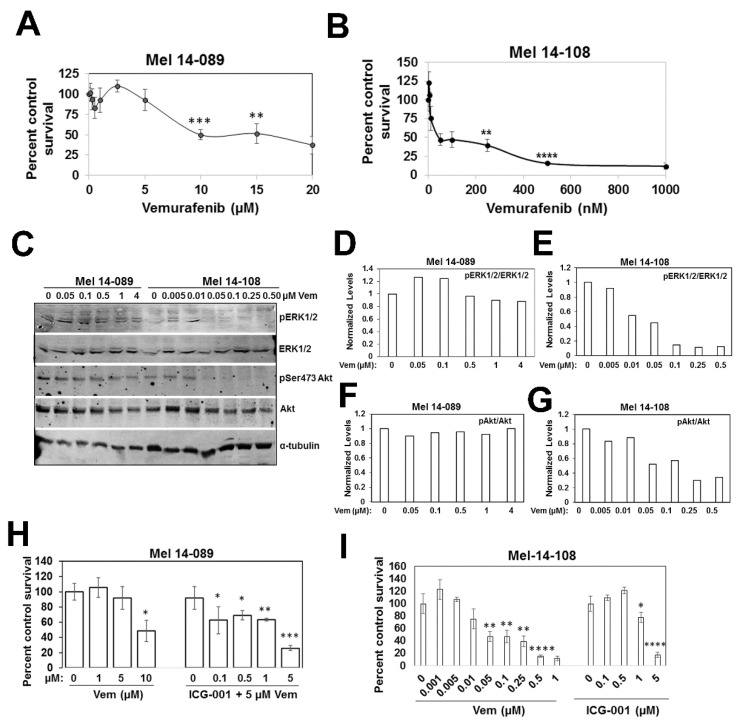
Vemurafenib sensitivity and ICG-001 effects on patient-derived melanoma cells with wild-type or mutant BRAF. (**A**,**B**) Evaluation of vemurafenib sensitivities of wild-type BRAF Mel 14-089 (**A**) and V600E BRAF Mel 14-108 (**B**) cells by MTT assays. Results are expressed as mean ± S.D. relative to control cell survival from two independent experiments each performed in quadruplicate. (**C**) Western blot analysis of the indicated proteins in Mel 14-089 and Mel 14-108 cells following overnight vemurafenib treatment, and (**D**–**G**) normalized levels of phospho-ERK1/2 and phospho-Akt in Mel 14-089 (**D**,**F**) and Mel 14-108 (**E**,**G**) cells. (**H**,**I**) ICG-001 treatment inhibits the survival of both patient-derived Mel 14-089 (**H**) and Mel 14-108 (**I**) melanoma cells. Results are expressed as mean ± S.D. relative to control cells from two independent experiments, each performed in quadruplicate. * *p* < 0.05; ** *p* < 0.01; *** *p* < 0.001, **** *p* < 0.0001.

To determine the clinical relevance of BRAF/ERK and β-catenin signaling, we conducted studies using two patient-derived brain metastatic melanomas, Mel 14-108 and Mel 14-089, that express *BRAFV600E* or wild-type BRAF, respectively. Consistent with its BRAF mutational status, MTT assays showed that Mel 14-108 cells are more sensitive to vemurafenib (IC50 ~50 nM) compared to Mel 14-089 cells with IC50 ~10 µM, and the remaining surviving Mel 14-089 cells remained refractory to doses up to 20 µM (Figure 4A,B). These data are corroborated by Western blot analysis that showed vemurafenib abrogated ERK1/2 phosphorylation in Mel 14-108 cells but had minimal effects in Mel 14-089 cells (Figure 4C–E). Concomitant with ERK1/2 inhibition, vemurafenib treatment also suppressed Akt Ser473 phosphorylation in Mel 14-108 cells while Akt phosphorylation was unaffected in Mel 14-089 cells (Figure 4C,F,G). These data suggest a common link between ERK1/2/Akt regulation and vemurafenib sensitivity in patient-derived melanomas. To test whether β-catenin/BRAF crosstalk is affected by BRAF mutational status, we analyzed the effects of ICG-001 on Mel 14-108 or Mel 14-089 cell survival by MTT assays. Despite differences in vemurafenib responses, ICG-001 treatment inhibited the proliferation of both patient-derived melanomas (Figure 4H,I). These data suggest a BRAF mutation-independent role for β-catenin in vemurafenib-refractory melanomas and that melanomas with mutant or wild-type BRAF could potentially benefit from inhibition of β-catenin signaling.

### 3.3. VemR Melanoma Cells Have Elevated Levels of Mitochondrial Mass and OXPHOS Activity

**Figure 5:**
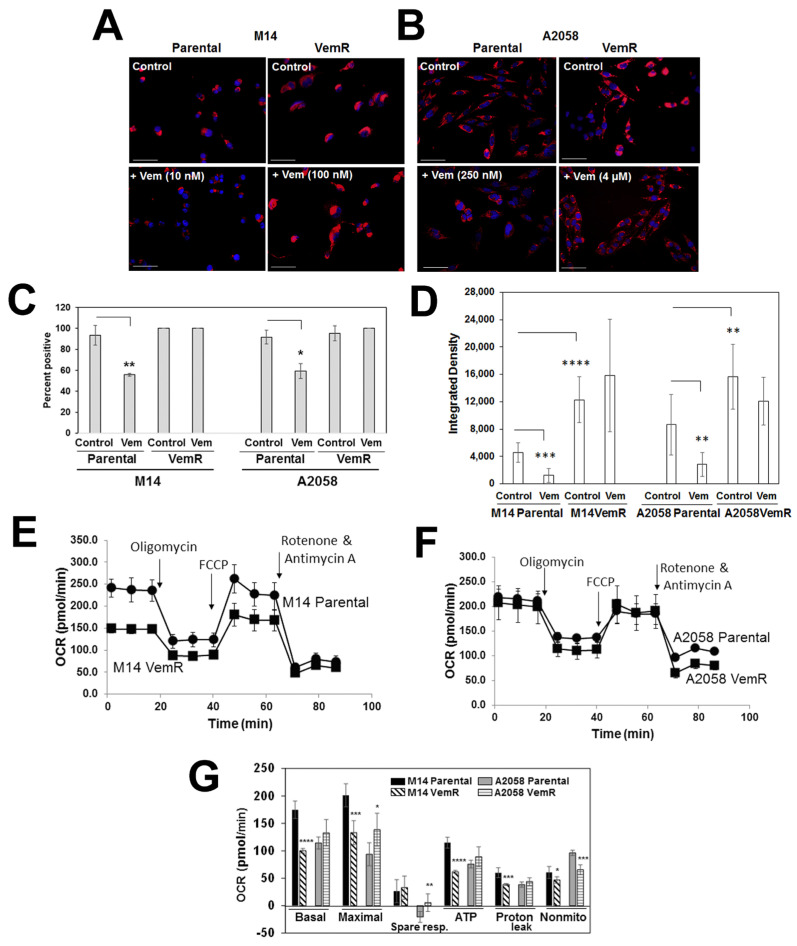
Vemurafenib-resistant melanoma cells have increased mitochondrial mass and spare respiratory capacity. (**A**,**B**) Mitotracker DeepRed staining of parental and VemR M14 and A2058 cells. Scale bar, 10 µm. (**C**) Percent of Mitotracker Red positive cells and (**D**) active mitochondrial area density determined using ImageJ. A minimum of 30–50 cells from three to five fields were scored, and the results are expressed as mean ± S.D. (**E**–**G**). Bioenergetics analysis of parental and VemR M14 (**E**) and A2058 (**F**) cells by Seahorse flux analyzer using a mito stress kit. (**G**) Summary data calculated from the curves in E and F. *, *p* < 0.05; **, *p* < 0.01; ***, *p* < 0. 001 ****; *p* < 0.0001.

WNT signaling functions in both glycolysis and mitochondrial respiration [28]. Since our data implicated β-catenin and MITF in adaptive VemR, we next determined if adaptive VemR involves metabolic rewiring to mitochondrial metabolism in our models. Mitochondrial mass and activity of parental and VemR cells were measured by staining with Mitotracker Deep Red FM, a mitochondrial potential-dependent dye. Vemurafenib treatment of parental M14 and A2058 cells resulted in significant decreases in the percentage of cells with active mitochondria (*p* < 0.05) and mitochondrial mass (*p* < 0.01) compared to untreated cells (Figure 5A–D). In contrast, M14 VemR and A2058 VemR cells displayed stronger Mitotracker Red staining intensities and mitochondrial mass that were unaffected by treatment with 100 nM or 5 µM vemurafenib, respectively (Figure 5A–D).

The oxygen consumption rate (OCR), an indicator of OXPHOS activity, was determined using the Seahorse XF Cell Mito Stress test under basal conditions and in response to sequential treatment with electron chain complex inhibitors (oligomycin, rotenone/antimycin A) and mitochondrial decouplers (FCCP). Mitochondrial functions were normalized to cell number and calculated based on the bioenergetics profiles that include basal respiratory capacity, maximal respiration, spare respiratory capacity, and ATP production. The results in Figure 5F,G show that both parental and VemR A2058 cells have similar levels of basal OCR. However, maximal respiration, an indicator of reserve or spare respiratory capacity (after injection of the uncoupler FCCP), was significantly higher in A2058 VemR (*p* = 0.027) cells compared to its parental counterpart. M14 VemR cells have significantly lower basal and maximal OCR compared to their parental counterparts (*p* < 0.001) (Figure 5E,G). However, although maximal respiration was lower in M14 VemR cells compared to parental cells, the spare respiratory capacity of M14 VemR cells was slightly higher, although not significantly, suggesting its potential for coping with drug-induced metabolic stress (Figure 5G). ATP-linked respiration was higher in A2058 VemR cells, reflecting its greater threshold of vemurafenib tolerance. These data are consistent with the greater Mitotracker Red staining intensities and mitochondrial mass in VemR cells and suggest that alterations in mitochondrial bioenergetics are commensurate with vemurafenib tolerance thresholds.

To gain insights into the regulation of OXPHOS levels by vemurafenib in parental and VemR M14 and A2058 cells, we evaluated the levels of subunits of OXPHOS complexes II-V by Western blot analysis. Among the four complexes, levels of UQCRC2 (ubiquinol-cytochrome c reductase), a core protein required for assembly of complex III, were marginally higher in both VemR models compared to their parental counterparts. COX II (complex IV subunit) levels were also marginally induced in A2058 VemR, and no discernible differences in SDHB (complex II subunit) or ATP5A (complex V subunit) protein levels between the parental and VemR cells were observed (Figure S4).

### 3.4. VemR Melanoma Cells Have Increased Abilities to Utilize Galactose as the Sole Energy Source

**Figure 6:**
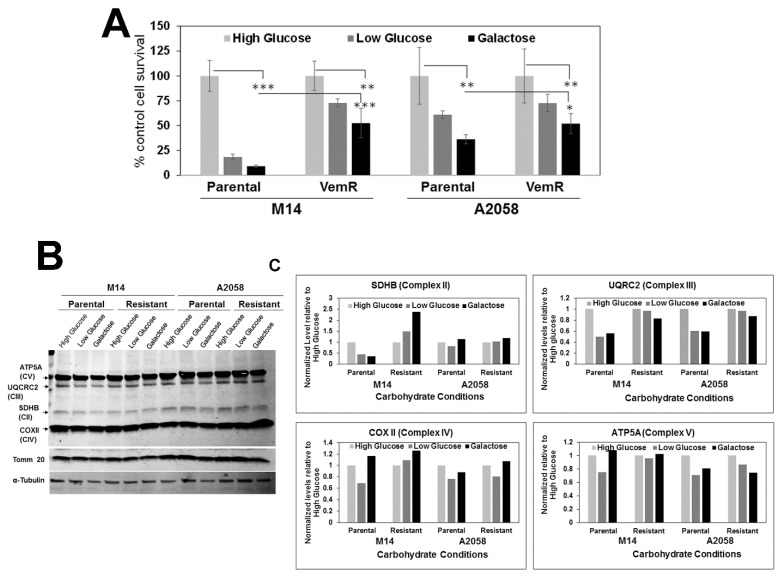
VemR melanoma cells display increased ability to utilize galactose as the energy source. (**A**) Survival analysis of parental and VemR M14 and A2058 cells under high-glucose, low-glucose, or galactose conditions by MTT assays. Results are expressed as mean ± S.D. relative to cell survival in high glucose from three independent assays, each performed in quadruplicate. *, *p* < 0.05; **, *p* < 0.01; ***, *p* < 0.001. (**B**) Western blot analysis of OXPHOS complex proteins and the (**C**) quantification of the levels of OXPHOS protein subunits normalized to Tomm20 and expressed relative to the high-glucose condition.

Our results thus far suggest that VemR acquisition involves a gain in mitochondrial activity. To confirm these data, we tested the impact of glucose vs. galactose as the only oxidizable substrate on survival and bioenergetics. Parental and VemR M14 or A2058 isogenic cells were cultured in medium containing 25 mM (high) glucose, 5 mM (low) glucose, or 25 mM galactose, and their survival under these conditions was assessed by MTT assays. A replacement of glucose with galactose forces cells to shift their energy source from glycolysis to mitochondrial oxidative phosphorylation [29]. Compared to parental M14 cells that were significantly growth inhibited in low-glucose or galactose media (*p* < 0.001), their VemR counterpart survived significantly better (~5-fold) under these conditions (*p* < 0.001), suggesting a gain or improvement in mitochondrial function. A2058 VemR cells showed similar increases in their ability to utilize galactose, albeit at a smaller magnitude (~2-fold), compared to their parental counterparts (*p* < 0.05; Figure 6A). Since A2058 VemR cells performed only slightly better than their parental counterparts under low-glucose or galactose conditions, we posit that the small differences in magnitude of metabolic shifts between the parental and VemR A2058 cells potentially reflect their intrinsically greater thresholds for vemurafenib tolerance. Since parental A2058 cells are inherently more tolerant of vemurafenib (IC50 250 nM) compared to parental M14 cells (IC50 10 nM), we speculate that parental A2058 cells are already utilizing both glycolytic and mitochondrial pathways for their energy needs, while M14 VemR cells may be forced to use mitochondria.

Next, we tested if the differences in glucose and galactose utilizations by parental vs. VemR isogenic melanoma models are associated with concomitant changes in OXPHOS complex II-V protein levels. Western blot analysis showed that UQCRC2 decreased ~50% in M14 and A2058 parental cells grown in galactose or low-glucose media compared to cells in high-glucose medium, whereas UQCRC2 levels remained elevated in VemR cells and were unaffected by galactose or low glucose (Figure 6B,C). Levels of SDHB, a subunit of complex II, were also decreased in parental M14 cells under galactose or low-glucose conditions and were upregulated in their VemR counterpart (Figure 6B,C). No differences in SDHB levels were seen in A2058 derivatives, and the levels of COX II and ATP5A levels constituting complexes IV and V, respectively, were not impacted (Figure 6B,C).

### 3.5. β-Catenin Signaling Promotes Mitochondrial Bioenergetics in VemR Melanoma Cells

**Figure 7:**
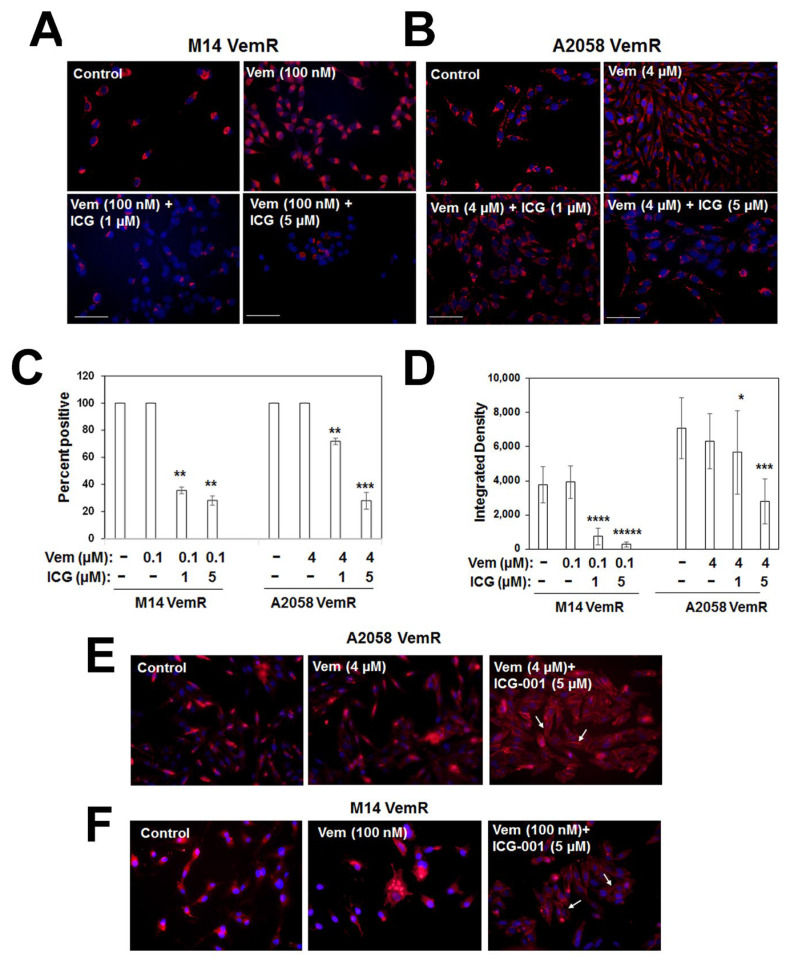
ICG-001 treatment decreases mitochondrial mass and activity in VemR melanoma cells. (**A**,**B**) MitoTracker DeepRed staining of VemR M14 and A2058 cells. Scale bar, 10 µm. (**C**) Percent of MitoTracker Red positive cells and (**D**) active mitochondrial density determined using ImageJ. Approximately 30–50 cells from 3–5 fields were scored and results expressed as mean ± S.D. *, *p* < 0.05, **; *p* < 0.01; ***, *p* < 0. 001; ****, *p* < 0.0001; *****, *p* < 0.00001. (**E**,**F**) Immunofluorescence staining of β-catenin in VemR A2058 and M14 cells treated with ICG-001. Original magnification 40×. Arrows in (**E**,**F**) indicate ICG-001 induced decreases in intracellular β-catenin and its relocalization to the cell surface.

Our data in Figure 1, Figure 2 and Figure 3 show that β-catenin signaling plays an important role in adaptive VemR development and that VemR development is associated with a gain in mitochondrial bioenergetics. To determine whether β-catenin activity is involved in the switch to mitochondrial metabolism, we analyzed the effects of ICG-001 on mitochondrial activity. While vemurafenib treatment did not alter Mitotracker Red staining intensities or the percentage of cells with active mitochondria in M14 VemR or A2058 VemR cells, treatment with ICG-001 significantly decreased the percentage (*p* < 0.01) and intensities of Mitotracker Red-stained (*p* < 0.001) cells (Figure 7A–D). ICG-001 treatment affected the survival of parental M14 as very few surviving cells were detected in the ICG-001/vemurafenib combination treatment groups. Parental A2058 cells responded similarly to ICG-001 as its VemR counterpart. Compared to control parental A2058 cells that showed intense punctate Mitotracker Red staining distributed throughout the cell and concentrated in filipodia, A2058 cells treated with a combination of 250 nM vemurafenib and 1 µM ICG-001 showed diffused Mitotracker Red staining or loss of staining in the filipodia (Figure S5). These data imply a role for Wnt/β-catenin signaling in parental cells and are consistent with basal TOP/Flash reporter activities in parental cells (Figure 3E). Immunofluorescence staining of β-catenin showed ICG-001 induced dramatic loss of intracellular β-catenin and relocation to the cell membrane in VemR cells (Figure 7E,F). Taken together with the TOP/Flash reporter and β-catenin-regulated gene expression data in Figure 3E–G, these data suggest that ICG-001 induced sensitization results from its inhibition of β-catenin activity.

**Figure 8:**
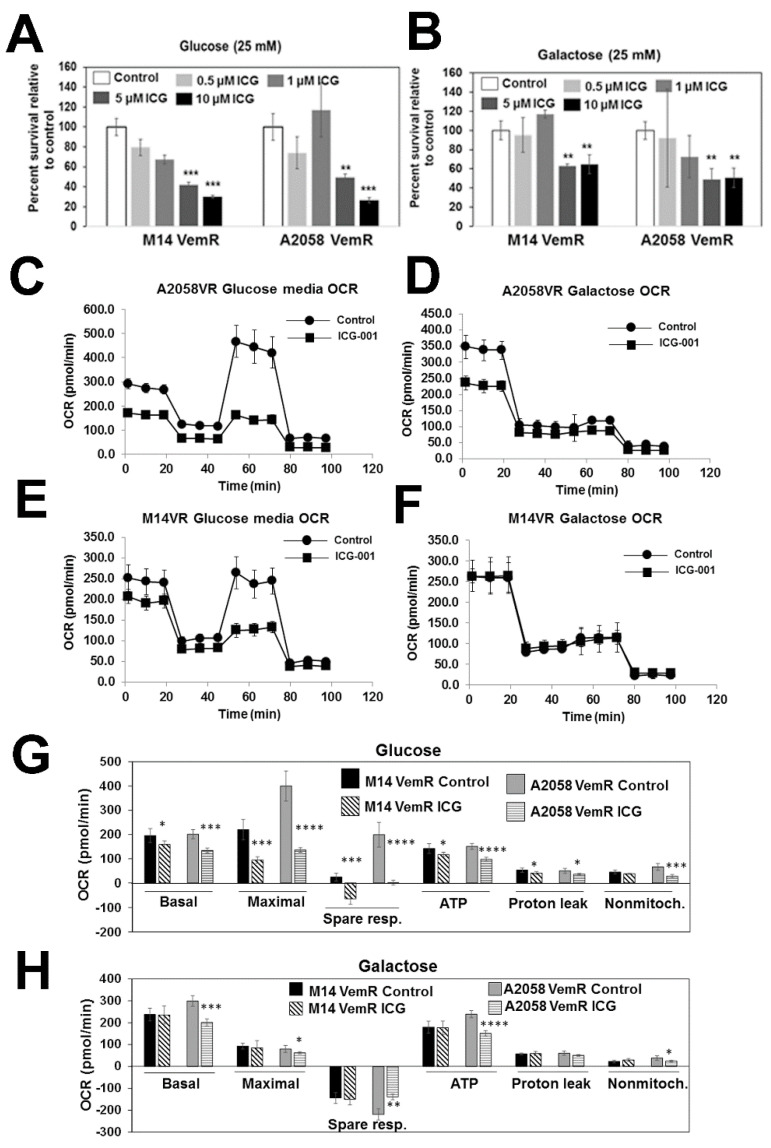
Vemurafenib-resistant melanoma cells maintain sensitivity to ICG-001 under high-glucose and galactose culture conditions. (**A**,**B**) Evaluation of ICG-001 sensitivities of VemR M14 and A2058 cells cultured in high-glucose or galactose media using MTT assays. Results are expressed relative to control mean ± S.D. from triplicate and two independent experiments. **, *p* < 0.01, ***, *p* < 0.001. (**C**–**F**) Bioenergetics analysis of VemR A2058 (**C**,**D**) and M14 (**E**,**F**) cells by Seahorse assay using mitochondrial stress kit under high glucose (**C**,**E**) and galactose (**D**,**F**). (**G**,**H**) Summary data calculated from the curves in (**C**–**F**). *, *p* < 0.05; **, *p* < 0.01; ***, *p* < 0. 001; ****, *p* < 0.0001.

Cells cultured in galactose rely mostly on OXPHOS, which makes them more susceptible to mitochondrial toxicants [30]. To determine if ICG-001 treatment will render different sensitivities to M14 or A2058 VemR cells grown under high-glucose or galactose conditions, cells cultured in glucose or galactose media were treated with 0.5–10 µM ICG-001, and cell survival was analyzed by MTT assays. A dose-dependent reduction in cell survival was observed with increasing concentrations of ICG-001 under both high-glucose and galactose conditions, suggesting involvement of β-catenin signaling in both glycolysis and OXPHOS pathways (Figure 8A,B). To isolate the involvement of β-catenin signaling in OXPHOS activity, we measured OCR of M14 and A2058 VemR cells treated with ICG-001 under glucose and galactose conditions. ICG-001 treatment significantly decreased basal and maximal OCR in both A2058 VemR (*p* < 0.001) and M14 VemR (basal, *p* < 0.05; maximal, *p* < 0.001) cells cultured in glucose media (Figure 8C,E,G). ICG-001 treatment similarly reduced basal (*p* < 0.001) and maximal (*p* < 0.05) respirations of A2058 VemR cells cultured in galactose media (Figure 8D,H), confirming involvement of β-catenin signaling in oxidative phosphorylation. M14 VemR cells grown in galactose or glucose media showed similar levels of basal OCR, which were inhibited similarly by mitochondrial ATP synthase inhibitor oligomycin (Figure 8E–H). These data suggest that M14 VemR cells have similar levels of basal OCR ascribed to ATP turnover under glucose and galactose conditions. However, in M14 VemR cells cultured in galactose, FCCP injection failed to stimulate the respiratory chain. It is interesting to note that while proton leak and non-mitochondrial consumptions of oxygen are decreased by ICG-001 under glucose in both M14 VemR and A2058 VemR cells (Figure 8G), they were unaffected by ICG-001 in both M14 VemR and A2058 VemR cells cultured in galactose media (Figure 8H). Since FCCP injection following oligomycin treatment fails to induce maximal respiration in cells grown under galactose, it suggests that respiration in these cells is controlled by oxidative phosphorylation. While ICG-001 treatment inhibited basal OCR in A2058 VemR cells (Figure 8D,H), it had no effect on the bioenergetics of M14 VemR cells grown in galactose (Figure 8F,H). These data suggest that β-catenin-dependent components required for oxygen consumption are probably compromised in M14 VemR cells cultured in galactose media. Analysis of ECAR, presented as absolute rates from a stable baseline, showed 5–10% increases in both VemR model cells compared to their parental counterparts (Figure S6A,B). Treatment with ICG-001 resulted in 1.8- to 2-fold decrease in ECAR in M14 VemR and A2058 VemR cells, respectively, that were grown in 25 mM glucose media. The ECAR in 25 mM galactose medium was only ~15–20% of what it was in 25 mM glucose medium, and while ICG-001 had no effect on ECAR in M14 VemR cells, it decreased ECAR in A2058 VemR cells cultured in galactose, indicating continued maintenance of glycolytic metabolism and involvement of the β-catenin pathway (Figure S6C,D).

### 3.6. Vemurafenib Resistance Development Is Associated with Alterations in Metabolic Pathways Involved with TCA Cycle and Pentose Phosphate Pathway

**Figure 9:**
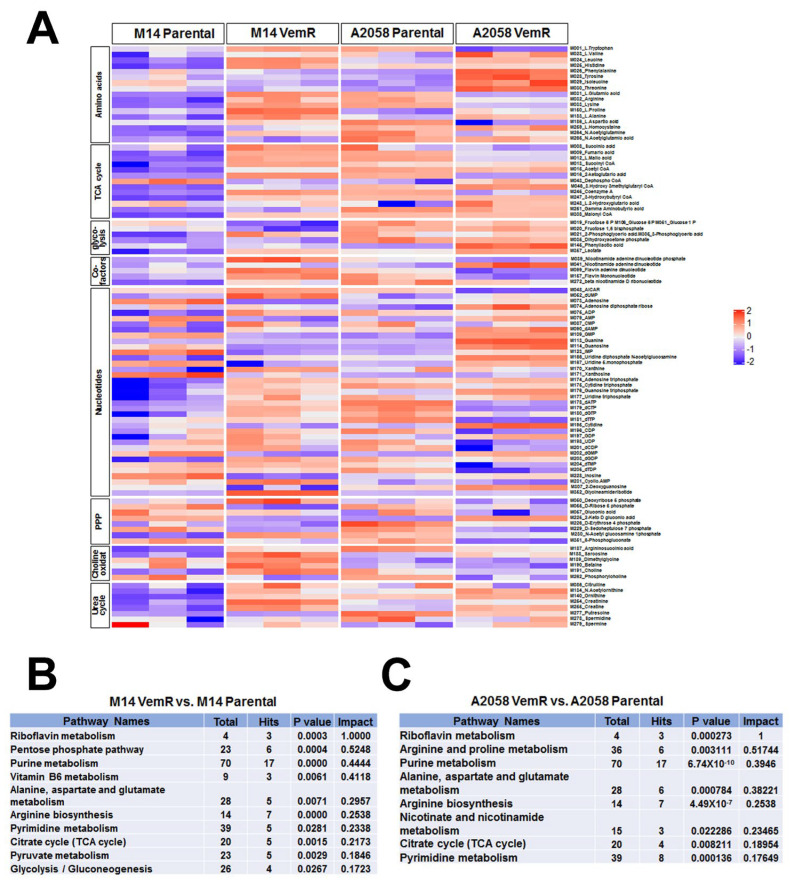
Acquisition of vemurafenib resistance alters the metabolome of melanoma cells. (**A**) A heatmap of targeted metabolomics data from isogenic parental and vemurafenib-resistant melanoma cells. Each column represents a sample, and each row represents the expression levels of a single metabolite. The color scale of the heatmap ranges from blue (low expression) to red (high expression). (**B**,**C**) Pathway analysis of top significantly altered metabolites and a metabolic pathway impact between M14 VemR and parental (**B**) and A2058 VemR vs. parental cells were evaluated by Metabolite Set Enrichment Analysis (MSEA) using MetaboAnalyst. A cutoff value of 0.1 for pathway impact score was used consistently across multiple comparisons to filter less important pathways.

To gain an overview of the metabolic differences between parental and VemR melanoma cells, we performed a comparative analysis of metabolites using an LC-MS/MS-based targeted metabolomics platform that measures 254 metabolites involved in major human metabolic pathways [20]. The steady-state levels of metabolites are shown in the heatmap (Figure 9A). Pathway analysis of differential metabolites in parental vs. VemR M14 and A2058 cells showed that metabolites involved in nucleotide (purine and pyrimidine biosynthesis), amino acid metabolism (alanine, aspartate, and glutamate metabolism; arginine biosynthesis), and the citric acid (TCA) cycle were significantly impacted by VemR acquisition in both models (Figure 9B,C). Metabolites involved in PPP were strongly impacted (impact score = 0.5248, *p* = 0.0004) by adaptive VemR in the M14 model (Figure 9B), whereas arginine/proline metabolism was significantly impacted (impact score = 0.51744, *p* = 0.003) in the A2058 model (Figure 9C). Similar analysis of metabolites in control and vemurafenib-treated patient-derived Mel 14-108 (*BRAFV600E*) and Mel 14-089 (BRAF wild-type) melanomas showed that vemurafenib treatment significantly impacted pathways related to glycine, serine, and threonine metabolism, PPP, NAD metabolism, pyruvate metabolism, arginine, glycolysis, and CoA biosynthesis only in BRAF mutant Mel 14-108 cells, while vemurafenib had no effect on these processes in BRAF wild-type Mel 14-089 cells (Figure S7 and Table S1A,B). These data confirm the selectivity of vemurafenib for mutant BRAF and mutant BRAF-driven metabolic pathways.

### 3.7. VemR Melanoma Cells Have Increased Ability to Metabolize Mitochondrial Substrates to Generate ATP

**Figure 10:**
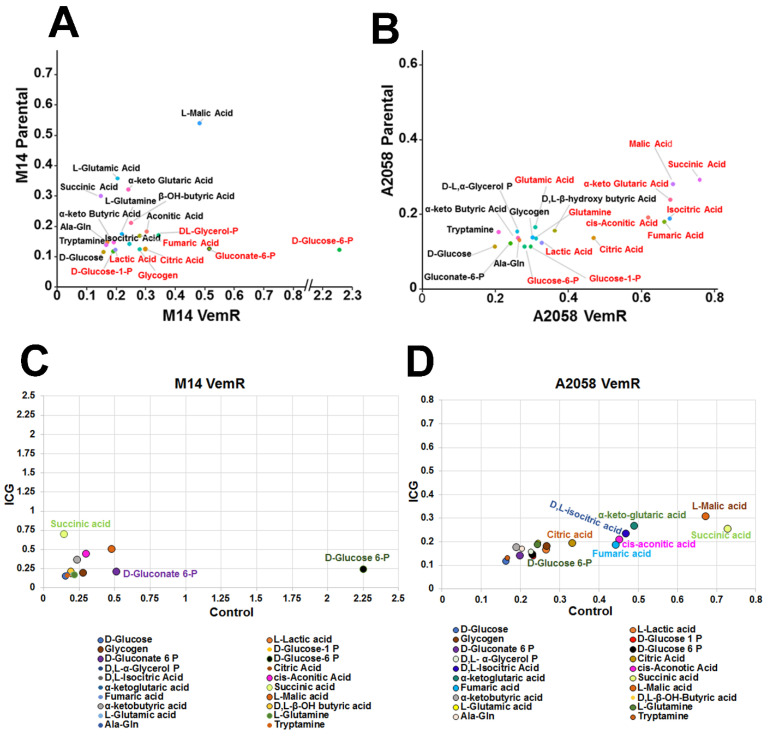
Mitochondrial metabolism and substrate preference analysis of vemurafenib-resistant melanoma cells and ICG-001 inhibition. (**A**) Parental and VemR M14 cells or (**B**) parental and VemR A2058 cells were plated at a density of 30,000 cells/well in duplicates, and the oxidation of a panel of cytoplasmic and mitochondrial substrates was assessed using the Biolog MitoPlate-S1 assay. Maximal oxidation of each substrate was determined, and data are represented in scatter plots to show the maximal rates of various substrates between parental and resistant cell lines. Each dot represents a unique substrate. Substrates marked in red indicate those preferentially metabolized by the respective model. (**C**,**D**) Metabolism of cytoplasmic and mitochondrial substrates by M14 VemR (**C**) and A2058 VemR (**D**) cells treated overnight with ICG-001. The data are represented in scatter plots to show the differences in maximal rates of substrate metabolism between control and ICG-001-treated VemR M14 and A2058 cells for the same substrate. Only substrates impacted by ICG-001 are labeled. Succinic acid, D-Gluconate 6-P and D-Glucose 6-P are impacted by ICG-001 in M14 VemR cells; and D-Glucose 6-P, Citric acid, Fumaric acid, D,L-isocitric acid, cis-Aconitic acid, Succinic acid, L-Malic acid and α-keto-glutaric acid are impacted by ICG-001 in A2058 VemR cells.

The metabolite analysis data in Figure 9 suggest an important role for mitochondria in VemR cells. To investigate the metabolic capacities and ATP generation, we assessed the mitochondrial functionality of parental and VemR counterparts using the MitoPlate S-1 assay, which employs microplates precoated with various NADH and FADH2-producing metabolic substrates [31,32]. We observed increases in metabolism of a variety of cytoplasmic (glycolytic), TCA cycle, and other mitochondrial substrates by M14 VemR and A2058 VemR cells relative to their corresponding parental counterparts (Figure 10A,B). However, A2058 VemR cells exhibited notably very high metabolic rates for TCA cycle substrates, citric acid, isocitric acid, aconitic acid, succinic acid, fumaric acid, malic acid, and α-keto-glutaric acid. The metabolic rate for these substrates was >2–3-fold higher in A2058 VemR cells compared to their parental control (Figure 10B). While cytoplasmic substrates, such as glycogen, glucose 1-phosphate, glucose 6-phosphate, glycerol phosphate, and lactic acid, were also metabolized at slightly higher rates in A2058 VemR cells compared to parental cells, the magnitudes of these metabolic rates were notably less compared to the usage of TCA cycle substrates (Figure 10B). Although the metabolic rates for the TCA cycle and other mitochondrial substrates were ~1.5-fold higher in M14 VemR cells compared to their parental cells, they were substantially lower than in A2058 VemR cells (Figure 10A). Most notably, in M14 VemR cells, the metabolic rates of PPP key substrates, glucose 6-phosphate, and gluconate-6-phosphate were ~15- and 3.6-fold, respectively, higher than their parental counterpart (Figure 10A), and were more pronounced than in A2058 VemR cells (Figure 10A). These data reveal enhanced metabolism via the mitochondrial and pentose phosphate pathways in adaptive VemR development. Since our data have shown an important role for β-catenin signaling in VemR development and metabolism, we determined the effect of ICG-001 on the metabolism of cytoplasmic and mitochondrial substrates using the MitoPlate S-1 assay. ICG-001 treatment selectively reduced the metabolic rates of glucose-6-phosphate and gluconate-6-phosphate by 9- and 2-fold, respectively, in M14 VemR cells compared to parental cells, while it had minimal effects on the utilization of other cytoplasmic or mitochondrial substrates (Figure 10C). Similar analysis of ICG-001 effects on A2058 VemR cells showed 1.5- to 2.8-fold decreases in metabolic rates of citric acid, isocitric acid, aconitic acid, succinic acid, fumaric acid, malic acid, and α-keto-glutaric acid but had little effect on glucose-6-phosphate and gluconate-6-phosphate utilization (Figure 10D). Taken together, the results from targeted metabolome and MitoPlate S-1 substrate utilization assays reveal a major role for β-catenin signaling in controlling the TCA cycle (a central hub in mitochondrial OXPHOS) in A2058 VemR cells and the PPP in M14 VemR cells.

### 3.8. Transcriptome Analysis Supports VemR-Associated Alterations in β-Catenin Signaling and Metabolic Pathways

**Figure 11:**
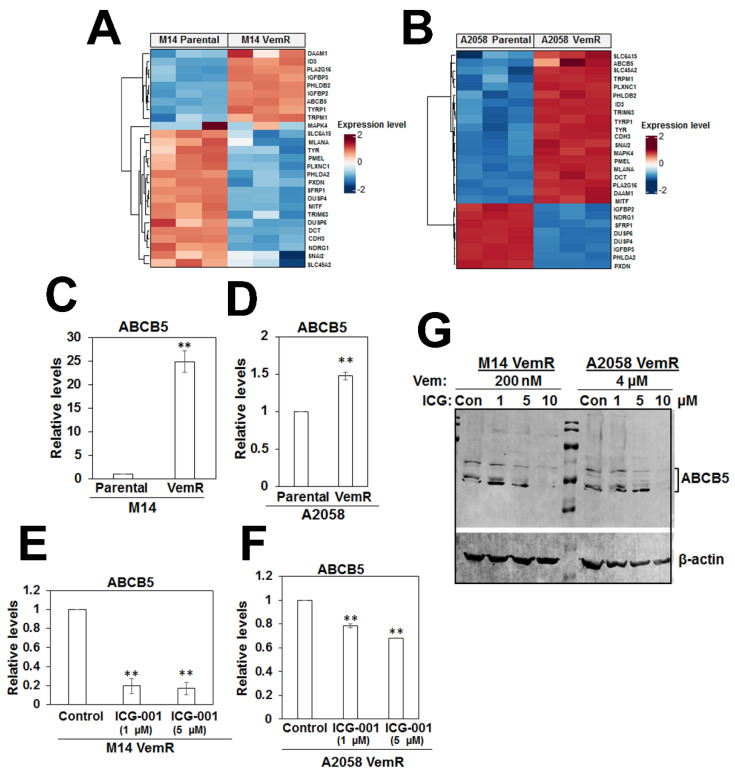
Canonical Wnt signaling-regulated gene expressions are upregulated in vemurafenib-resistant melanoma cells. (**A**,**B**) A heatmap of RNA-seq data comparing Wnt signaling-regulated gene expressions in parental and VemR M14 (**A**) or parental and VemR A2058 cells. (**C**–**F**) Quantitative RT-PCR analysis of ABCB5 gene expression in parental and VemR M14 (**C**) and A2058 (**D**) cells, and the regulation of ABCB5 expression by ICG-001 treatment in M14 VemR (**E**) and A2058 (**F**) cells. (**G**) Western blot analysis of ABCB5 and regulation by ICG-001 in VemR M14 and A2058 cells. **, *p* < 0.01.

To analyze the molecular underpinnings of VemR, we characterized the global gene expression profiles of parental and VemR M14 and A2058 cells by whole transcriptome sequencing, and statistically significant differentially expressed genes (DEGs) were subjected to iPathway guide analysis to identify the affected processes. Volcano plots and meta-analysis using Venn diagram revealed 2158 and 2163 statistically significant DEGs (FDR ≤ 0.05) in M14 VemR vs. M14 and A2058 VemR vs. A2058 cells, respectively, of which 867 genes were commonly expressed in M14 VemR and A2058 VemR groups (Figure S8A–E). Among the 867 genes, 282 and 200 genes, respectively, were upregulated and downregulated in both VemR models (Figure S8D). Pathway analysis of the affected DEGs revealed enrichment of genes associated with 34 pathways in M14 VemR and A2058 VemR cells (Figure S8E) and encompassed pathways associated with EGFR tyrosine kinase resistance, PPAR signaling, MAPK signaling, Ras signaling, cytokine receptor interaction, neuroactive ligand receptor interaction, and PI3K-Akt signaling pathways (Figure S8F). Hierarchical cluster analysis showed clear separations of DEGs between parental and VemR M14 and A2058 groups (Figure 11A,B) and identified upregulation of several genes associated with Wnt signaling in both VemR models (Figure 11A,B). Among the Wnt/β-catenin-regulated genes overexpressed in both VemR cells were TRPM1, ID3, ABCB5, TYRP1, PLA2G16 [33], and PHLDB2 [34]; whereas, SFRP1 and NDRG1 [35] negative regulators of Wnt/β-catenin signaling were downregulated in both M14 VemR and A2058 VemR cells compared to their parental counterparts. ID3 (inhibitor of differentiation) and ABCB5 (subfamily B, member 5) transporter (a member of the ATP-binding cassette (ABC) superfamily) overexpression is reported in VemR melanomas [36]. ABCB5 has been identified as a marker of melanoma-initiating cells [37], and its expression is induced by β-catenin [38]. Real-time RT-PCR analysis confirmed overexpression of ABCB5 in M14 VemR and A2058 VemR cells compared to their parental counterparts (Figure 11C,D). Treatment with ICG-001 significantly decreased ABCB5 gene expression (*p* < 0.01) and protein in both VemR models (Figure 11E–G), confirming its regulation by the Wnt/β-catenin pathway. The expression of canonical Wnt signaling-regulated genes MLANA, MITF, DCT, TYR, and PMEL (premelanosome) were upregulated in A2058 VemR cells; however, these were downregulated in M14 VemR cells, suggesting potential reprogramming of pigmentation program in M14 cells upon acquisition of VemR (Figure 11A,B). Pathway analysis of transcripts in patient-derived BRAF mutant Mel 14-108 melanoma showed similar enrichment of pathways associated with cytokine–cytokine receptor interaction, ECM receptor interaction, and neuroactive ligand receptor interaction as in BRAF mutant M14 and A2058 models, whereas pathways distinct from those regulated by mutant BRAF, viz., cell cycle, DNA replication, Fanconi anemia, and DNA repair pathways, were enriched in patient-derived wild-type BRAF Mel 14-089 melanoma cells (Table S2).

**Figure 12:**
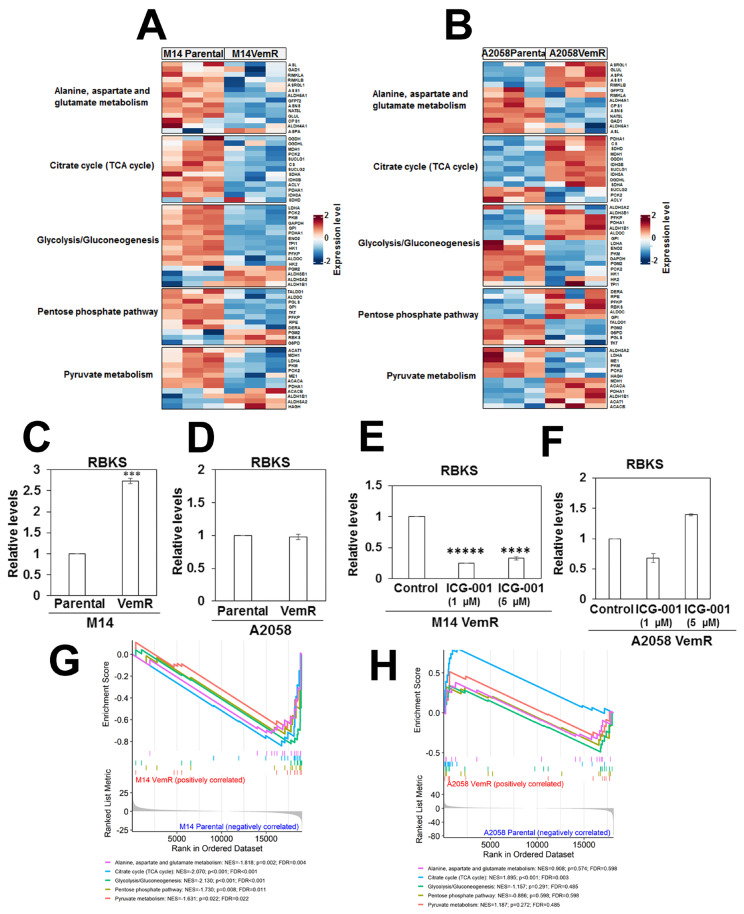
Transcript-metabolic pathway relationship. (**A**,**B**) A heatmap of genes regulating metabolic pathways impacted by VemR in M14 (**A**) and A2058 (**B**) isogenic pairs. (**C**–**F**) Quantitative RT-PCR analysis of RBKS gene expression in parental and VemR M14 (**C**) and A2058 (**D**) cells, and regulation of RBKS expression by ICG-001 treatment in M14 VemR (**E**) and A2058 (**F**) cells. ***, *p* < 0. 001; ****, *p* < 0.0001; *****, *p* < 0.00001. (**G**,**H**) Gene set enrichment analysis (GSEA) plots of five representative pathway gene sets between M14 VemR and M14 parental groups (**G**) and (**H**) between A2058 VemR and A2058 parental cells.

The relationship between VemR-impacted metabolic pathways and their regulatory mRNAs was analyzed in parental and VemR M14 and A2058 cells by integrating the expressions of genes involved in the impacted metabolic pathways. Key PPP genes Glucose 6 phosphate dehydrogenase (G6PD, a rate limiting enzyme in the PPP), Ribokinase (RBKS, a PfkB family member of carbohydrate kinases responsible for ribose phosphorylation) and Phosphoglucomutase 2 (PGM2, involved in ribose and deoxyribose phosphate catabolism) were elevated in M14 VemR cells compared to their parental cells (Figure 12A). In contrast, several genes associated with the TCA cycle (CS, MDH1, OGDH, SUCLG1) were enriched in A2058 VemR cells compared to their parental counterparts (Figure 12B). The higher expression of RBKS in M14 VemR cells is consistent with the elevated levels of 2-deoxy ribose 5-phosphate in M14 VemR cells (Figure 9A). Overexpression of RBKS gene expression was validated by real-time RT-PCR analysis, which showed a 2.75-fold increase (*p* < 0.001) in RBKS levels in M14 VemR cells compared to the parental cells (Figure 12C). Treatment with ICG-001 significantly decreased RBKS gene expression in M14 VemR cells (*p* < 0.0001), suggesting regulation by the Wnt/β-catenin pathway (Figure 12E). RBKS gene expression was not significantly different between parental and VemR A2058 cells (Figure 12D) and was unaffected by ICG-001 treatment (Figure 12F). These data corroborate the metabolome (Figure 10) and metabolite utilization data from the MitoPlate S1 assay (Figure 11) and indicate strong roles for the TCA cycle and the PPP in VemR development of A2058 and M14 cells, respectively. Taken together, our data suggest that the acquisition of VemR and survival involve a key role for Wnt/β-catenin-induced alterations in pathways associated with glucose catabolism.

Gene set enrichment analysis (GSEA) was conducted on genes corresponding to five metabolic pathways (Figure 12G,H). All five pathways were significantly enriched in the M14 parental group, with negative normalized enrichment scores (NES) indicating downregulation in M14 VemR relative to the parental counterpart. Glycolysis/gluconeogenesis (NES = −2.130, *p* < 0.001, FDR < 0.001) and citrate cycle (TCA cycle) (NES = −2.070, *p* < 0.001, FDR < 0.001) pathways showed the highest negative NES values, followed by alanine, aspartate, and glutamate metabolism (NES = −1.818, *p* = 0.002, FDR = 0.004), PPP (NES = −1.730, *p* = 0.008, FDR = 0.011), and pyruvate metabolism (NES = −1.631, *p* = 0.022, FDR = 0.022) (Figure 12G). The citrate cycle pathway was the most enriched in the A2058 VemR cells compared to their parental counterpart, with a positive NES (NES = 1.895, *p* < 0.001, FDR = 0.003) (Figure 12H).

## 4. Discussion

Metabolic plasticity, a key feature of melanoma, plays a major role in the development of drug resistance and tumor aggressiveness. However, no studies to date have analyzed the relationships between vemurafenib endurance levels and the specific metabolic pathways melanomas co-opt to ensure survival. To uncover how drug endurance levels drive metabolic shifts, we performed an integrative analysis of metabolome and transcriptome data from isogenic human metastatic *BRAFV600E* melanoma models with variant vemurafenib tolerance thresholds (M14 VemR and A2058 VemR with IC50 100 nM and 5 µM, respectively) and patient-derived brain metastatic melanomas with wild-type (Mel 14-089) or V600E mutant (Mel 14-108) BRAF. Our analysis showed that VemR models, regardless of their drug tolerance differences, displayed resistance to MEK inhibitors and retained vulnerability to Wnt/β-catenin signaling inhibition despite activating distinct metabolic pathways.

The hallmark of metabolic reprogramming by cancer cells is a shift towards glycolysis or the Warburg effect as the key energy production mechanism [39], and a trend towards elevated mitochondrial biogenesis and oxidative phosphorylation has been observed in *BRAFV600E* melanomas that develop drug resistance [11,40]. Metabolic reprogramming is regulated by both canonical and noncanonical Wnt signaling [41] in many tumor types, including melanoma [42], and Wnt signaling regulates the Warburg effect by inducing expression of key regulators of glycolysis [43]. Studies suggest an important role for PTEN function in mediating Wnt/β-catenin-induced upregulation of mitochondrial dynamics [44,45]. In this context, it is interesting to note that despite differences in PTEN status, both PTEN wild-type M14 and PTEN null A2058 VemR cells exhibit increased mitochondrial activity, bioenergetics, and Wnt/β-catenin activity, and treatment with the Wnt/β-catenin antagonist ICG-001 similarly reduces mitochondrial activity and OCR in both VemR models. These data suggest a PTEN-independent role of Wnt/β-catenin signaling in the regulation of VemR, which is supported by our data showing minimal differences in LY294002 sensitivities between the parental and VemR counterparts.

Mitochondrial stress assays using Seahorse showed that although M14 VemR cells have significantly lower basal and maximal OCRs compared to their parental counterparts, their spare or reserve respiratory capacity is slightly higher than their sensitive counterparts. The spare respiratory capacities of A2058 VemR cells are also higher than their corresponding parental counterparts, and analysis of OXPHOS levels showed upregulation of UQCRC2, a component of complex III, in both resistant models. These data provide support for a common role for mitochondria in coping with drug-induced metabolic stress. To verify switch to mitochondrial metabolism, the OCRs of VemR cells were compared under glucose and galactose conditions since galactose increases a cell’s dependence on mitochondrial ATP production [46,47]. Similar levels of basal OCR were seen under both sugar conditions, suggesting efficient usage of the mitochondria by both A2058 and M14 VemR cells for ATP production. While treatment with ICG-001 dramatically reduced ATP levels and basal and spare respiratory capacities under both glucose and galactose conditions in A2058 VemR cells, this inhibition occurred only under glucose culture conditions in M14 VemR cells. These data reveal differences in Wnt/β-catenin pathway dependence, with A2058 VemR cells exhibiting greater reliance on the Wnt/β-catenin pathway for OXPHOS compared to M14 VemR cells. Both isogenic models of VemR have slightly higher or similar levels of ECAR compared to their sensitive counterparts, indicating that the glycolytic flux of VemR cells remains stable despite the switch to mitochondrial metabolism. Although galactose itself should not inhibit glycolysis flux, culturing in galactose media resulted in a ~5-fold decrease in ECAR compared to cells in glucose. The reason for this is unclear; however, it is possible that the coerced reliance on mitochondrial ATP production could redirect pyruvate away from lactic acid conversion into the mitochondria for OXPHOS. In such a scenario, this should decrease lactate production. On the contrary, our data from targeted metabolite analysis and MitoPlate S-1 assays show higher levels of lactate, suggesting a more complex OCR–ECAR relationship under galactose treatment. Since ICG-001 treatment reduced ECAR under both sugar conditions in A2058 VemR cells and only under glucose in M14 VemR cells, it implicates dual roles for the Wnt/β-catenin pathway in modulating glycolytic and OXPHOS metabolism.

RNA-seq analysis revealed that numerous genes regulated by the Wnt/β-catenin pathway (ABCB5, TYRP1, ID3, TRPM1) are upregulated while the negative Wnt/β-catenin regulator SFRP1 is downregulated in both M14 and A2058 VemR cells compared to their parental counterparts. Studies have reported upregulation of ID3 (inhibitor of differentiation 3, a helix-loop-helix TF superfamily member) in melanomas of patients treated with a BRAF inhibitor, and silencing ID3 resulted in enhanced sensitization of melanoma to vemurafenib [48]. RNA-seq analysis (further validated by qRT-PCR and protein analysis) also identified overexpression of ABCB5, a member of the ATP-binding cassette (ABC) transporter superfamily, in both VemR models. ABC transporters facilitate the ATP-driven efflux of drugs across cellular membranes [49,50] and are associated with multidrug resistance in several cancers, including melanoma [51]. ABCB5 is a marker of melanoma aggressiveness, multidrug resistance, and stemness [33,52,53], and has been reported to induce unique modifications of metabolism in melanoma-initiating cell lines [54]. The ABCB5 gene encodes several isoforms, including ABCG5FL, a full transporter, ABCB5β, a half transporter, and heterodimers of ABCB5β with ATPase activity [55]. In melanoma, its expression is activated by Wnt/β-catenin and MITF [39], and as expected, ICG-001 treatment suppressed ABCB5 mRNA as well as expression of ABCB5 immunoreactive isoforms in both our VemR models.

Metabolomics and mitochondria-function based MitoPlate S-1 assays identified upregulation of TCA cycle metabolism in both M14 and A2058 VemR cells. However, the metabolic rates of TCA cycle substrate utilization, as measured by MitoPlate S-1 assays, were considerably higher in A2058 VemR cells compared to M14 VemR cells, and suggest that the net effects of these genes could lead to hyperactivation of the TCA cycle. Although both M14 VemR and A2058 VemR cells undergo mitochondrial metabolic reprogramming, the magnitude of this metabolic shift differed greatly and correlated with the vemurafenib tolerance thresholds, as melanomas with greater levels of de novo or acquired drug tolerance (such as A2058) were better at exploiting mitochondrial pathways for their bioenergetic needs. Consistent with the increased reliance of A2058 VemR cells on the mitochondria for energy production, integration of metabolomics pathway analysis data with RNA-seq analysis confirmed overexpression of several genes associated with the TCA cycle (CS, PDHA1, MDH1, OGDH, IDH3B, IDH3A, SUCLG1, OGDHL) in A2058 VemR cells. The two most important products produced from the PPP branch are ribose 5-phosphate, used to make DNA and RNA, and the antioxidant nicotinamide adenine dinucleotide phosphate (NADPH) for nucleotide synthesis and maintenance of redox homeostasis [56] to facilitate cancer cell survival under drug-induced metabolic stress [57]. Our results show that among the genes associated with PPP, G6PD, RBKS (critical for synthesis of ribose 5-phosphate), and PGM2 (regulator of ribose and deoxyribose phosphate catabolism) [58,59] are increased in M14 VemR cells, which is consistent with the robust utilization of PPP substrates glucose 6-phosphate and gluconate 6-phosphate, and increases in levels of deoxy ribose 5-phosphate. MitoPlate S-1 assays conducted with A2058 and M14 VemR cells treated with β-catenin antagonist showed suppression of the TCA cycle and PPP substrate utilizations in A2058 VemR and M14 VemR cells, respectively. An important role for Wnt/β-catenin in conferring chemoresistance via induction of G6PD expression and activation of PPP has been reported [60]. Real-time RT-PCR analysis of RBKS confirmed its overexpression in M14 VemR cells, and consistent with MitoPlate S-1 data, RBKS gene expression is inhibited by ICG-001. Our data from metabolomic analysis of patient-derived metastatic Mel 14-108 melanoma with comparable vemurafenib sensitivity profiles as M14 cells showed similar vemurafenib-induced metabolic pathway perturbations (PPP, nicotinamide metabolism, pyrimidine metabolism, glycine/serine/threonine metabolism), supporting the relationship between low vemurafenib tolerance and PPP activation. Since these pathways were not perturbed by vemurafenib in wild-type BRAF expressing Mel 14-089 cells confirm the selectivity of the drug for mutant BRAF (Figure S7 and Table S1A,B). These results imply an important role for the Wnt/β-catenin pathway in modulating distinct metabolic pathways in VemR cells and indicate that melanoma cells with low vemurafenib tolerance limits divert metabolism to the PPP, while melanoma cells with high drug tolerance thresholds choose mitochondrial metabolism for surviving drug-induced metabolic stress. There are limitations to this study as our data were obtained primarily from in vitro studies, albeit from a range of experimental systems. Additional functional and in vivo studies using more melanoma models with variant vemurafenib sensitivities are required for assessing the therapeutic efficacy of targeting metabolic-specific pathways identified in our study.

## 5. Conclusions

Our results show that Wnt/β-catenin signaling plays a major role in controlling metabolic plasticity and drug resistance, and that targeting this signaling could produce strong interference with these activities in BRAF inhibitor- and MEK inhibitor-resistant melanomas. Our data also show that drug tolerance thresholds play a direct role in driving metabolic shifts towards specific metabolic routes and that an enhanced understanding of this relationship could better inform the selection of metabolic pathway-specific therapies for the treatment of vemurafenib-resistant melanomas.

## Data Availability

The datasets generated for this study can be found in the Gene Expression Omnibus (GEO) (http://ncbi.nlm.nih.gov/geo/, sunmitted on 13 December 2024, undated on 7 January 2025, remain private until 12 December 2025), database through accession # GSE284250.

## Supplementary Materials

The following supporting information can be downloaded at: https://www.mdpi.com/article/10.3390/cells14120923/s1, Figure S1: Sensitivity analysis of BRAF mutant human metastatic melanoma cells to vemurafenib; Figure S2: Sensitivity analysis of parental melanoma cells to MEK and PI3K inhibitors; Figure S3: Sensitivity analysis of VemR melanoma cells to MITF inhibitor ML329; Figure S4: OXPHOS protein analysis in parental and VemR melanoma cells; Figure S5: ICG-001 effects on mitochondrial activity in parental melanoma cells; Figure S6: ECAR rates of parental and VemR M14 and A2058 cells and effect of ICG-001 under glucose and galactose conditions; Figure S7: Impact of vemurafenib on metabolome of melanoma patient derived cells Mel-14-108 (BRAF mutant) and Mel-14-089 (wild type BRAF); Figure S8: Vemurafenib resistance acquisition alters the transcriptome of melanoma cells; Table S1: Metabolic pathways impacted by vemurafenib in melanoma patient derived cells expressing mutant BRFAF (Mel-14-108) or wild type BRAF (Mel-14-089); Table S2: Pathway analysis of transcripts show distinct enrichment of pathways in patient derived melanoma cells with mutant BRAF (Mel 14-108) vs. wild type BRAF (Mel 14-089) cells.
