# Supplementary material for: Inter-Relationship Between Melanoma Vemurafenib Tolerance Thresholds and Metabolic Pathway Choice
Source: Cells. 2025 Jun 18;14(12):923. doi: 10.3390/cells14120923 (PMC12191169; doi:10.3390/cells14120923)
Supplement: Supplementary file 1 [file cells-14-00923-s001.zip › cells-3633445-supplementary.pdf]

Supplementary Data

Figure S1. Sensitivity analysis of BRAF mutant human metastatic melanoma cells to vemurafenib. (A,B) Clonogenic analysis of M14 (A) and A2058 (B) cells following treatment with the indicated concentrations of vemurafenib. (C,D) Visual depiction of wells captured from M14 (C) or A2058 (D) cells using GelCount Oxford Optronix. The red box in panel D, represents quadruplicates of A2058 cells treated with 10 or 50 μM vemurafenib or controls, and the green box in panel D, represents triplicate wells treated with 100, 250 or 500 μM vemurafenib. The purple box in the bottom right corner of panel D represents A2058 cells treated with 1 μM vemurafenib. Results are expressed as mean ± S.D. (percent of control colony formation efficiency) from three independent experiments. *P<0.05; **P<0.01; ***P<0.001; ****P<0.0001.

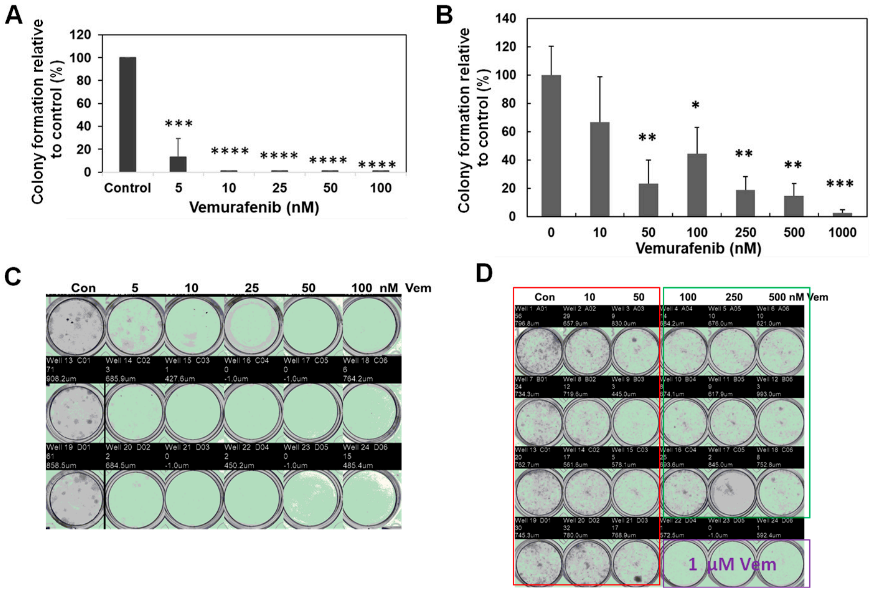

Figure S2. Sensitivity analysis of parental melanoma cells to MEK and PI3K inhibitors. Cells were treated with the indicated doses of U0126 or LY294002.

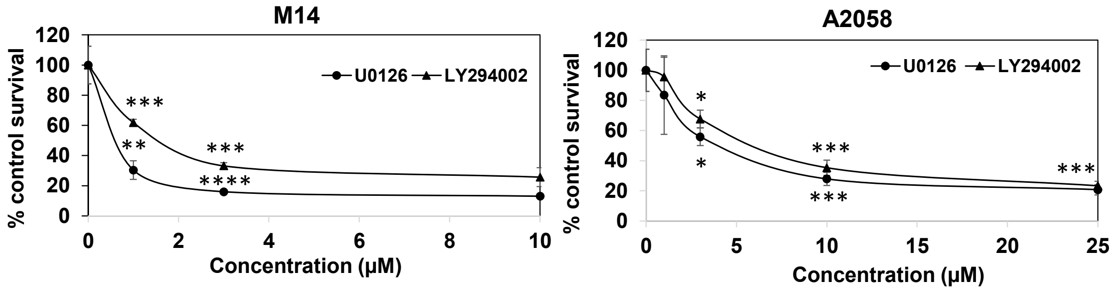

Figure S3. Sensitivity analysis of VemR melanoma cells to MITF inhibitor ML329. Cells were treated with the indicated concentrations of ML329.

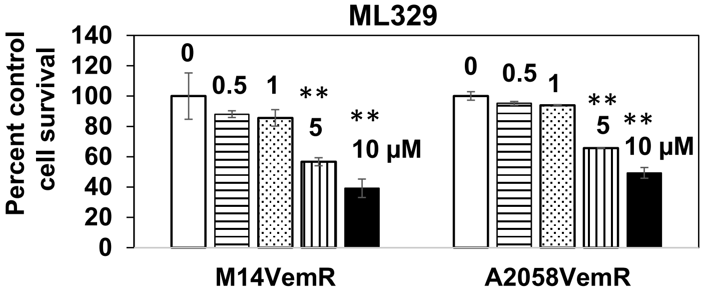

Figure S4. OXPHOS protein analysis in parental and VemR melanoma cells. (A, B) Western blot analysis of subunits of OXPHOS complexes in M14 and A2058 isogenic pairs treated with the indicated concentrations of vemurafenib. Quantification of the levels of OXPHOS protein subunits normalized to Tomm20 and expressed relative to control in M14 (C-I) and A2058 (D-J) isogenic cells.

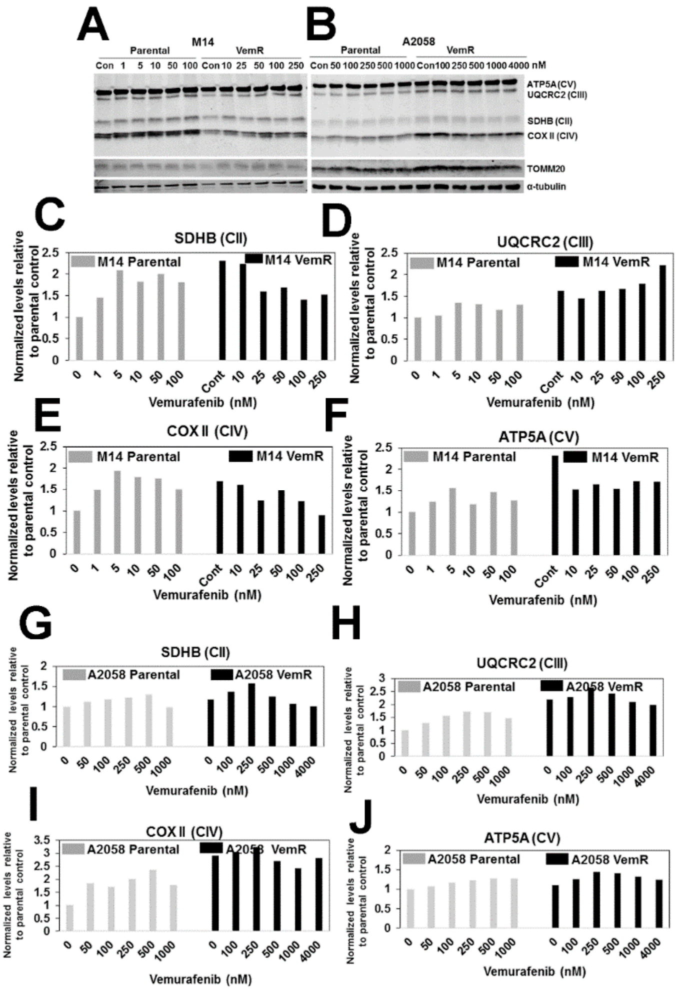

Figure S5. ICG-001 effects on mitochondrial activity in parental melanoma cells. Parental M14 or A2058 cells were treated overnight with a combination of 1 μM ICG-001 and 10 nM (M14) or 250 nM (A2058) Vem (vemurafenib) and cells were stained with MitoTracker DeepRed. Original magnification ×100. Control parental A2058 cells show intensely stained puncti and concentration of mitochondria in the filipodia (long arrows), while cells treated with Vem+ICG-001 show diffuse staining and loss of staining in the filipodia (arrow heads).

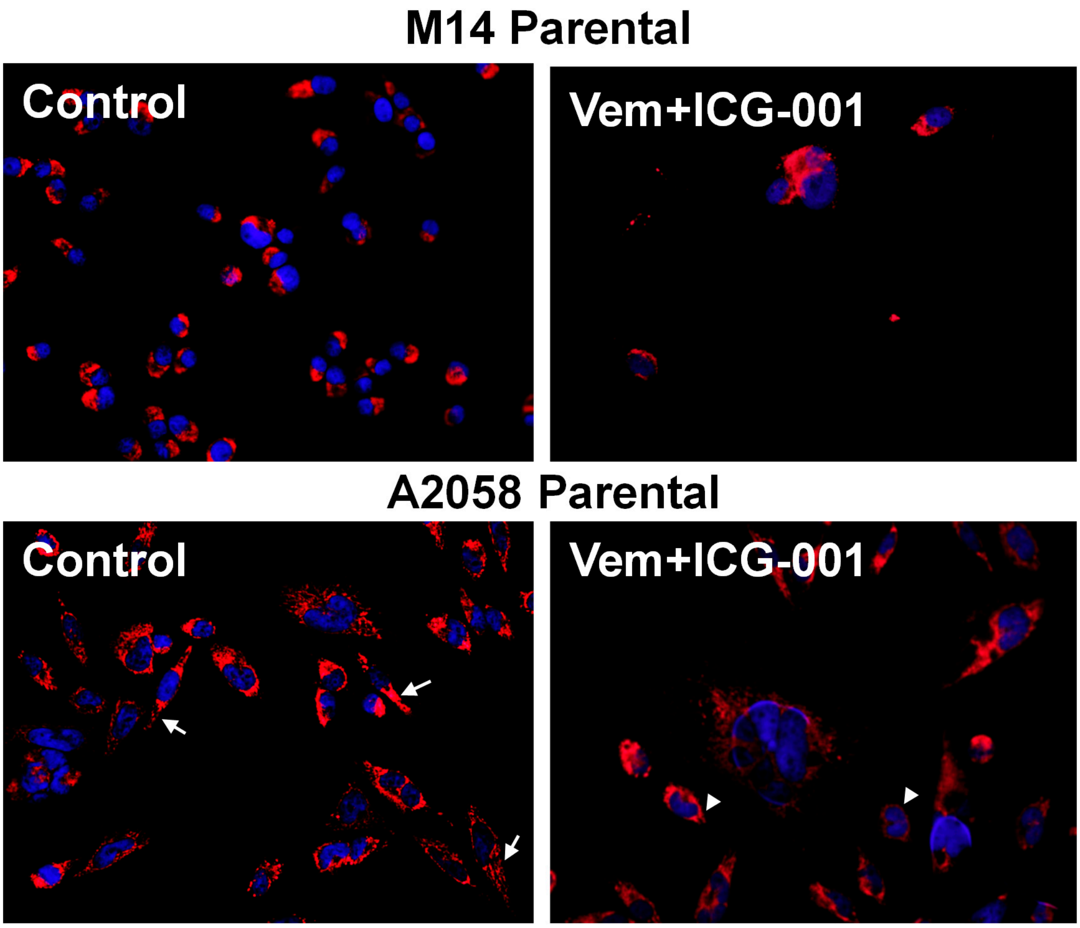
M14 Parental
Control
Vem+ICG-001
A2058 Parental
Control
Vem+ICG-001

Figure S6. ECAR rates of parental and VemR M14 and A2058 cells and effect of ICG-001 under glucose and galactose conditions. (A) Comparisons of ECAR rates of isogenic M14 and A2058 parental and VemR cells. (B) ICG-001 effects on ECAR under glucose and galactose conditions.

A

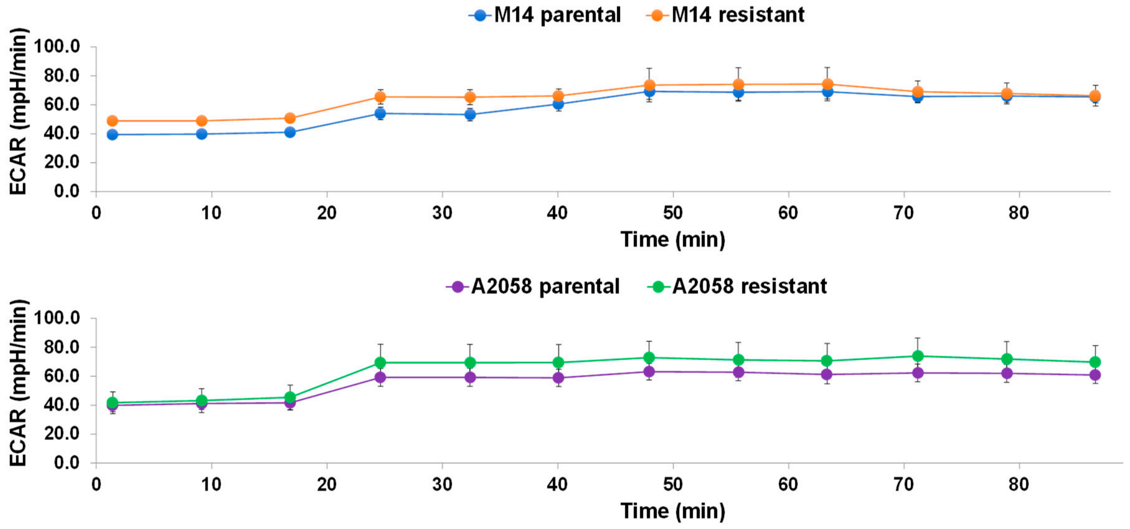

B

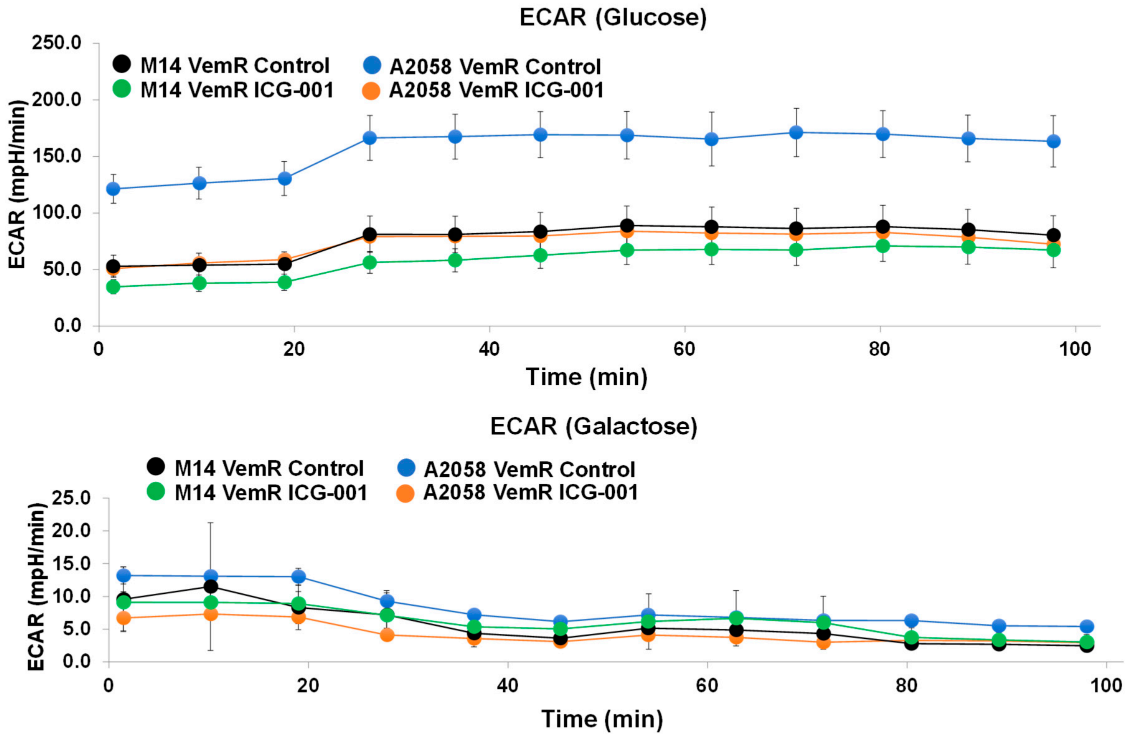

Figure S7. Impact of vemurafenib on metabolome of melanoma patient derived cells Mel-14-108 (BRAF mutant) and Mel-14-089 (wild type BRAF). .

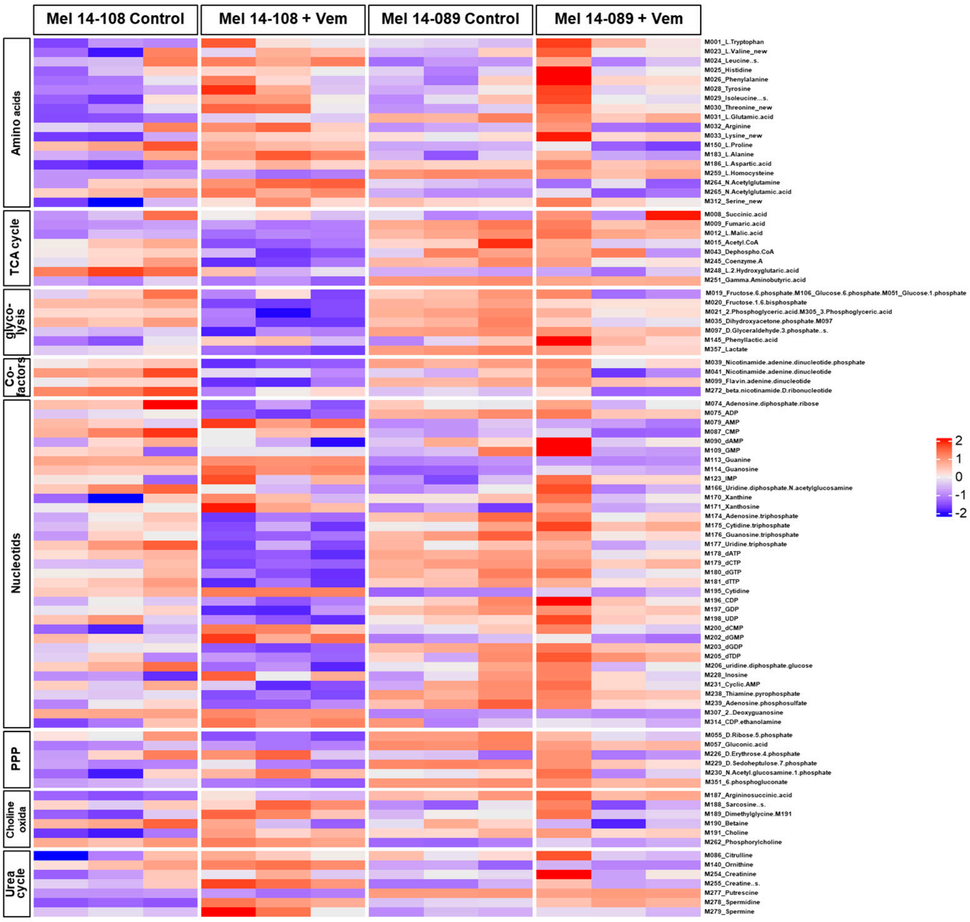

Figure S8. Vemurafenib resistance acquisition alters the transcriptome of melanoma cells. (A,B) Volcano plots of RNA-seq data comparing VemR vs. parental M14 (A) or VemR vs. parental A2058 (B) cells. Total number and differentially expressed genes (DEGs) genes 14756 and 3025, respectively (M14 VemR vs. M14 Parental), and 14079 and 3030, respectively (A2058 VemR vs. A2058 Parental) measured at 5% FDR and fold-change (FC) of ≥2. (C) Venn diagram of DEGs and (D) directional distribution of DEGs in M14 VemR vs. parental, and A2058 VemR vs. parental cells. (E) Meta analysis of pathways enriched in VemR M14 and A2058 cells, and (F) VemR impacted canonical pathways identified by iPathway Guide.

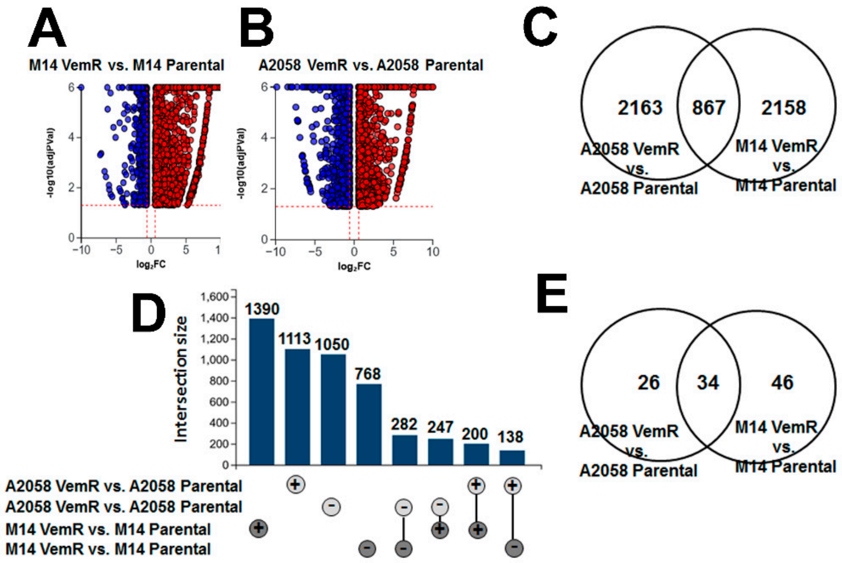

F

Pathway	A2058-VemR vs. A2058-Parental (p value)	M14-VemR vs. M14-Parental (p value)
EGFR tyrosine kinase inhibitor resistance	0.022827	0.000967
PPAR signaling pathway	0.041301	0.007768
MAPK signaling pathway	0.000203	0.001331
Ras signaling pathway	0.000125	0.018511
Rap1 signaling pathway	0.000551	0.007156
Calcium signaling pathway	0.001904	3.20E-08
Cytokine-cytokine receptor interaction	2.40E-07	3.69E-08
Neuroactive ligand-receptor interaction	1.9E-06	1.12E-08
PI3K-Akt signaling pathway	1.02E-06	8.13E-08
Vascular smooth muscle contraction	0.041617	0.002771
TGF-beta signaling pathway	0.000616	0.01217
Focal adhesion	2.94E-07	0.004178
ECM-receptor interaction	2.38E-06	0.004328
Cell adhesion molecules	7.75E-06	0.00035
Complement and coagulation cascades	0.002451	0.003262
Hematopoietic cell lineage	0.000103	0.017313
Regulation of actin cytoskeleton	0.001772	0.001418
Aldosterone synthesis and secretion	0.012668	0.003794
Cortisol synthesis and secretion	0.010542	0.016785
Proximal tubule bicarbonate reclamation	0.003037	0.026204
Pancreatic secretion	0.019415	0.038643
Protein digestion and absorption	3.66E-05	7.75E-06
Bile secretion	0.001595	0.009966
Mineral absorption	0.025012	0.000955
Malaria	0.007695	0.001784
Amoebiasis	0.00094	0.031214
Pathways in cancer	1.4E-05	0.035947
Transcriptional misregulation in cancer	0.000156	0.000649
Proteoglycans in cancer	0.008415	0.011636
Melanoma	0.015735	0.03431
Hypertrophic cardiomyopathy	0.000344	0.000103
Arrhythmogenic right ventricular cardiomyopathy	8.13E-06	0.000183
Dilated cardiomyopathy	4.47E-05	0.000165
Fluid shear stress and atherosclerosis	0.00046	0.033454

Table S1. Metabolic pathways impacted by vemurafenib in melanoma patient derived cells expressing mutant BRFAF (Mel-14-108) or wild type BRAF (Mel-14-089). Pathway analysis of top significantly altered metabolites and metabolic pathway impact were evaluated by Metabolite Set Enrichment Analysis (MSEA) using MetaboAnalyst. A cut-off value of 0.1 for pathway impact score was used consistently across multiple comparisons to filter less important pathways.

A Mel 14-108 Control vs. Vemurafenib

Pathway Names	Total	Hits	P value	Impact
Pantothenate and CoA biosynthesis	20	3	0.026026	0.35715
Glycine, serine and threonine metabolism	33	6	0.0005401	0.28885
Glutathione metabolism	28	5	0.0017827	0.28461
Pentose phosphate pathway	23	3	0.03772	0.28124
Nicotinate and nicotinamide metabolism	15	4	0.0011028	0.26623
Pyrimidine metabolism	39	8	2.29E-05	0.25961
Arginine biosynthesis	14	3	0.0095558	0.2335
Purine metabolism	70	16	1.22E-10	0.18448
Pyruvate metabolism	23	3	0.03772	0.16621
Glycolysis / Gluconeogenesis	26	5	0.0012547	0.11815

B Mel 14-089 Control vs. Vemurafenib

Pathway Names	Total	Hits	P value	Impact
Glutathione metabolism	28	2	0.010161	0.26315
Arginine and proline metabolism	36	2	0.016537	0.11395

Table S2. Pathway analysis of transcripts show distinct enrichment of pathways in patient derived melanoma cells with mutant BRAF (Mel 14-108) vs. wild type BRAF (Mel 14-089) cells.

Pathway	Mel 14-108 vs. Vem treated (p value)
Cytokine-cytokine receptor interaction	9.784E-07
Viral protein interaction with cytokine receptor interaction	6.923E-04
Rheumatoid arthritis	9.496E-04
Neuroactive ligand-receptor interaction	0.0001
Transcriptional misregulation in cancer	0.005
ECM-receptor interaction	0.008
Vascular smooth muscle contraction	0.008
cAMP signaling pathway	0.010

Pathway	Mel 14-089 vs. Vem treated (p value)
Cell cycle	1.384E-07
DNA replication	7.754E-06
Fanconi anemia pathway	8.939E-05
P53 signaling pathway	1.797E-04
Cellular senescence	2.063E-04
PI3K-Akt signaling pathway	8.922E-04
microRNAs in cancer	0.001
Mismatch repair	0.001
ECM-receptor interaction	0.002
